# Differentiation of human induced pluripotent stem cells into cardiac valve cells using 2D and 3D differentiation protocols

**DOI:** 10.1186/s13287-026-05132-z

**Published:** 2026-07-02

**Authors:** Z. Farzaneh, A. Brückner, B. K. Fleischmann, S. Rieck

**Affiliations:** Institute of Physiology 1, Medical Faculty, University of Bonn, Bonn, Germany

**Keywords:** Human induced pluripotent stem cells, Differentiation, Endocardial cells, Valve endothelial-like cells, Endothelial-to-mesenchymal transition

## Abstract

**Background:**

Given the high relevance of human cardiac valve disease, recent research aims to differentiate human induced pluripotent stem cells (hiPSCs) into valve endothelial-like cells (VELCs) and, through endothelial-to-mesenchymal transition (EndMT), into valve interstitial-like cells (VILCs).

**Methods:**

Here, we modified a 2D differentiation protocol demonstrating that VEGF can serve as the sole driver for differentiating cardiac progenitor cells (CPCs) into VELCs. Next, we utilized the so-called GiWi protocol (inhibition of glycogen synthase kinase, followed by inhibition of the Wnt pathway) to derive VELCs from 3D endocardial spheres. To this aim, hiPSCs were first differentiated into cardiac progenitor (CP) spheres using CHIR99021 (12 µM) and IWP2 (5 µM). Subsequent treatment with E8 medium containing high-dose FGF2 (100 ng/ml) resulted in endocardial spheres enriched for VELCs. For EndMT induction, endocardial spheres were MACS-sorted for PECAM1^+^ VELCs and transdifferentiated into ACTA2^+^/CDH5^−^ VILCs using TGFβ1 (200 ng/ml).

**Results:**

Using VEGF as main driver to differentiate CPCs into VELCs in 2D, the generated VELCs appeared stable over time, can be maintained in vitro and transdifferentiated into ACTA2^+^ VILCs using FGF2 (100 ng/ml) and TGFβ1 (50 ng/ml). Besides, our novel 3D differentiation protocol yielded endocardial spheres, which were highly enriched with VELCs, as shown by the expression of GATA4 (≈ 83%), PECAM1 (≈ 69%), and nuclear NFATC1 (≈ 76%), along with typical functional characteristics such as network formation, LDL uptake and the ability to undergo EndMT.

**Conclusions:**

Overall, we developed a 2D differentiation protocol that produces stable VELCs and a high percentage of VILCs upon EndMT induction. We also established a new, efficient, and cost-effective protocol for the 3D differentiation of endocardial spheres to better mimic a physiologically relevant environment, thereby enabling improved maturation.

**Supplementary Information:**

The online version contains supplementary material available at 10.1186/s13287-026-05132-z.

## Background

Aortic valve stenosis (AVS) is a severe heart disease affecting 0.3 to 0.5% of the population [1]. AVS can be either congenital (e.g. narrowed aortic valves, often bicuspid) or acquired (e.g. calcific degeneration). In industrialized countries, degenerative calcifying AVS is predominantly observed [2], but little is known about its onset and early pathophysiological features, due to the lack of symptoms at this stage. Moreover, phenocopying AVS in small animals is challenging due to technical limitations in in vivo access and monitoring, as well as differences from humans. Therefore, alternative models are required to obtain a more mechanistic understanding of the biology underlying human AVS.

For the investigation and better understanding of congenital and acquired AVS, endocardial cells, a specialized type of endothelial cells that form the endocardium of the heart, play a critical role. Valve endothelial cells (VECs) are a subtype of endocardial cells, and vital components of cardiac valves, as they cover the surface of the valve leaflets and have pleiotropic functions, such as regulating blood coagulation, nutrient transport, and immune cell infiltration [3]. Moreover, their migration to the inner layer of the valves during heart development, their role in forming the cardiac cushions, their transition to valve interstitial cells (VICs) through endothelial-to-mesenchymal transition (EndMT), and their potentially detrimental role in calcification and stenosis are important for AVS development.

Human induced pluripotent stem cells (hiPSCs) are a suitable source of human endocardial cells and could offer insights into the mechanisms governing EndMT and their role in AVS development in vitro. Given the recent strong interest in investigating AVS, initial studies have proposed protocols to differentiate hiPSC-derived VEC-like cells (VELCs) and probed their further transdifferentiation into VIC-like cells (VILCs) [4–9]. The protocols use either cardiac progenitor cells (CPCs) or cardiac/cardiogenic mesoderm as an intermediate stage, followed by specification into VELCs. Interestingly, the different strategies pursued to obtain this intermediate stage varied greatly, with either BMP10 [6], CHIR99021, or Wnt3a being employed to activate the Wnt pathway [4, 5, 7, 8, 10], or alternatively, the so-called GiWi protocol, which inhibits glycogen synthase kinase, followed by inhibition of the Wnt pathway [4]. 

To generate hiPSC-derived endocardial cells in vitro, we first tested a monolayer (2D) protocol [7], in which hiPSCs were differentiated into CPCs followed by their specification into VELCs using different growth factors. When we observed low stability and spontaneous transdifferentiation in the generated VELCs, we modified the protocol and identified VEGF as the primary driver of endocardial specification, resulting in stable VELCs. However, recent efforts in the hiPSC field focus on three-dimensional (3D) differentiation strategies, as this approach is thought to create a more physiological microenvironment for complex cell-cell interactions, similar to an in vivo setting compared to 2D monolayer protocols [11], and is more suitable for upscaling in high-throughput drug discovery or human tissue engineering [11]. To accomplish this, using chemically synthesized small molecules offers advantages, because these molecules are inexpensive, easy to produce, and can be specifically targeted in a dose-dependent, rapid, and reversible way. Therefore, we used the chemically defined GiWi protocol to differentiate endocardial spheres into 3D cultures containing a high percentage of VELCs. Thus, we have developed a 2D differentiation protocol for the stable production of hiPSC-derived VELCs, as well as a 3D protocol for creating endocardial spheres using small molecules and growth factors. This enables the generation of large quantities of authentic VELCs in vitro and their transdifferentiation into VILCs, thereby supporting the study of EndMT.

## Methods

### Cell culture media and supplements

hiPSC growth medium: StemMACS™ iPS Brew XF (Miltenyi Biotec, #130-104-368); E6 medium: Essential 6™ medium (Thermo Fisher Scientific, # A1516501); RPMI/B27 medium: RPMI 1640 (Thermo Fisher Scientific, #21875034) + 1x B27 supplement minus vitamin A (Thermo Fisher scientific, #12587010); E8 medium: Essential 8™ medium (Thermo Fisher Scientific, #A1517101); DPBS (Thermo Fisher Scientific, #14190144), DPBS^++^: DPBS with calcium and magnesium (Thermo Fisher Scientific, #14040117); 0.5 M EDTA (Thermo Fisher Scientific, #15575020); Trypsin-EDTA 0.05% (Thermo Fisher Scientific, #25300054); Matrigel (Corning, #354234) diluted 1:100 in knockout DMEM (Thermo Fisher Scientific, #10829018); BMP4 (Peprotech, #120-05); CHIR99021 (Cayman Chemicals, #13122); FGF2 (Peprotech, #100-18B); IWP2 (Tocris, #3533); purmorphamine (Tocris, #4551); SB431542 (Cayman Chemical, #13031); TGFβ1 (Peprotech, #100 − 21); VEGF-165 (Peprotech, #100 − 20); Wnt3a (Peprotech, #315 − 20), Y27632 (Selleckchem, #S1409).

### Cell lines

Human valve endothelial and interstitial cells (VECs and VICs) were obtained from Lonza (#00225975 or # 00225974) and only used for RNA isolation. The human induced pluripotent stem cell (hiPSC) lines iLB C16bm-s16 (https://hpscreg.eu/cell-line/UKBi015-B) and iLB C133bm-s4 (https://hpscreg.eu/cell-line/UKBi013-A) have been provided by the Cell Programming Core Facility of the University Bonn. iLB C16bm-s16 was used for all experiments, whereas iLB C133bm-s4 was only used to verify the 3D differentiation protocol.

### Cultivation of hiPSCs

hiPSCs were cultivated on Matrigel-coated plates in hiPSC growth medium. The cells were passaged when they reached 70%-80% confluence. Then, they were detached as cell clumps using 0.5 mM EDTA in DPBS for 3–4 min., and re-seeded at an appropriate ratio (1:8 to 1:12) in growth medium supplemented with 10 µM Y27632. 24 h after passaging, the medium was replaced with hiPSC growth medium without Y27632 and then replaced daily.

### Monolayer (2D) differentiation of hiPSCs into valve endothelial-like (VELCs) and valve interstitial-like cells (VILCs)

**Fig. 1:**
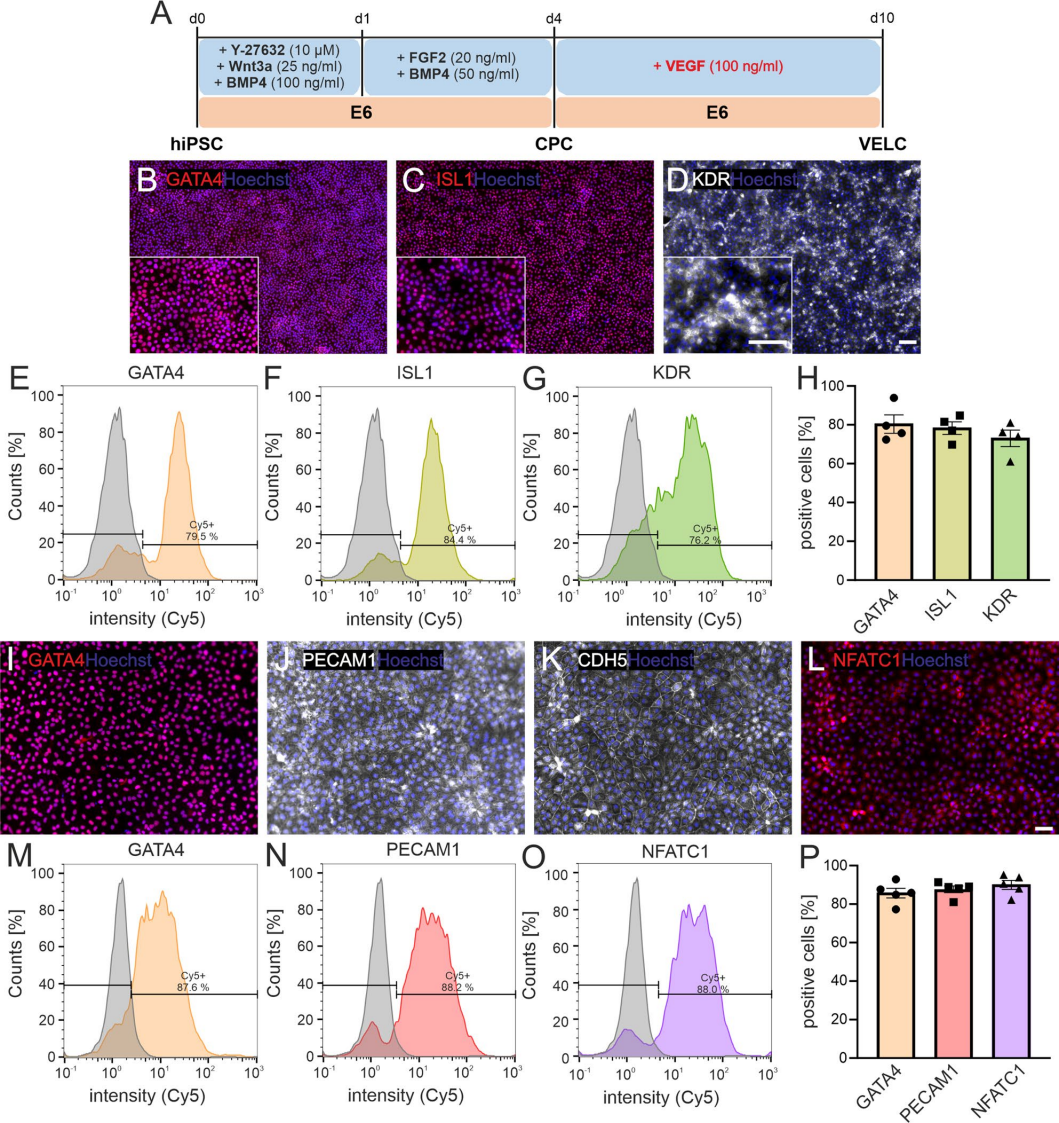
2D differentiation protocol for the generation of endocardial cells. **A** Human induced pluripotent stem cells (hiPSCs) were first differentiated into cardiac progenitor cells (CPCs), followed by further specification into valve endothelial-like cells (VELCs). **B-D** Immunofluorescence staining of CPCs at day 4 (d4) of differentiation; scale bar = 100 μm. **E**–**H** Flow cytometry histograms (**E**–**G**) and quantification (**H**) for GATA4, ISL1, and KDR expression in CPCs at d4 (*n* = 4). **I**–**L** Immunofluorescence staining of VELCs at day 10 (d10) of differentiation; scale bar = 50 μm. **M**–**P** Flow cytometry histograms (**M**–**O**) and quantification (**P**) for GATA4, PECAM1, and NFATC1 (single stainings) expression in VELCs at d10 (*n* = 4). Data are shown as mean ± SEM. Statistical analysis was performed using one-way ANOVA; * *p* ≤ 0.05, ** *p* ≤ 0.01, *** *p* ≤ 0.001, **** *p* ≤ 0.0001

Initially, the already published differentiation protocol from Cheng et al. [7] was used to differentiate hiPSC-derived VELCs and VILCs (Suppl. Figure 1A). This protocol was modified (Fig. 1A). Briefly, hiPSCs were detached using 0.5 mM EDTA in DPBS, collected in E6 medium supplemented with 25 ng/ml Wnt3a, 100 ng/ml BMP4, and 10 µM Y27632, and seeded with a density of 5 × 10^4^ cells/cm^2^ onto Matrigel-coated plates (day 0). From day 1 to day 4 of differentiation (d1-d4) the cells were cultivated in E6 medium supplemented with 20 ng/ml FGF2 and 50 ng/ml BMP4 with daily medium change. For further differentiation into VELCs, cells were dissociated on d4 with 0.05% Trypsin-EDTA for 3–5 min to obtain single cells, then resuspended in E6 medium supplemented with 100 ng/ml VEGF and 10 µM Y27632, and seeded at 1 × 10^5^ cells/cm^2^ on Matrigel-coated plates. From day 5 to day 10 of differentiation (d5-d10), cells were cultivated in E6 medium supplemented with 100 ng/ml VEGF, with medium change every other day. To convert VELCs into VILCs, endothelial-to-mesenchymal transition (EndMT) was induced by culturing cells in E6 medium supplemented with 100 ng/ml FGF2 and 50 ng/ml TGFβ1 for 6 days (d10-d16). Control cells were treated with 100 ng/ml VEGF for the same period.

### Three-dimensional (3D) differentiation of hiPSC-derived endocardial cells

In order to differentiate hiPSCs to VELCs and VILCs under 3D conditions, we adopted the already published protocol from Fonoudi et al. with modifications [12] (Fig. 3A): hiPSCs were dispersed as cell clumps using 0.5 mM EDTA in DPBS and transferred to low-adherence bacterial plates (Greiner, 628161) with approximately 10^6^ cells in 5 ml/dish to allow aggregate formation. Differentiation was initiated in 4-5-day-old hiPSC aggregates (hiPSC spheres, d0) by cultivation in RPMI/B27 medium containing 12 µM CHIR99021 for one day. On day 1 of differentiation (d1), aggregates were collected in the center of the plate by gentle swirling, washed with DPBS^++^, and cultivated for one additional day in RPMI/B27 medium without additives. On days 2 and 3 (d2-d3), the aggregates were treated with 5 µM IWP2 in RPMI/B27 medium, resulting in cardiac progenitor (CP) spheres at day 4 (d4). For further differentiation into endocardial spheres, the CP spheres were washed on d4 with DPBS^++^, then transferred to E8 medium and cultured for an additional 6 days with medium change every other day.

EndMT induction was either performed directly in endocardial spheres at day 10 of differentiation (d10) or in enriched PECAM1^+^ cells derived from dissociated endocardial spheres. For the direct EndMT induction, endocardial spheres were treated with E6 medium supplemented with 200 ng/ml TGFβ1 for 6 days. The control group received E8 medium without any growth factors. For the EndMT induction in isolated cells, the endocardial spheres were first dissociated using STEMdiff™ Cardiomyocyte Dissociation Kit (Stemcell Technologies, #05025) following the manufacturer’s protocol and then enriched for PECAM1 (CD31) using an anti-CD31 PE-conjugated antibody (Miltenyi Biotec, #130-117-313), anti-PE microbeads (Miltenyi Biotec, #130-118-495) and a QuadroMACS separator (Miltenyi Biotec, #130-090-976) with LS columns (Miltenyi Biotec, #130-042-401). Enriched PECAM1^+^ cells were seeded with a density of 1 × 10^4^ cells/cm^2^ on Matrigel-coated plates in E8 medium supplemented with 5 µM Y27632. 24 h after seeding, the medium was replaced with E8 medium without Y27632, and then every other day until the cells reached confluence. To induce EndMT, the cells were then cultivated in E6 medium containing 200 ng/ml TGFβ1 for 6 days.

### Differentiation of cardiac spheres

Beating cardiac spheres were differentiated as described elsewhere [12]. Briefly, hiPSCs that reached 80% confluence, with medium changes every other day, were detached with 0.5 mM EDTA in DPBS and seeded at a density of 10^6^ cells in 5 ml of hiPSC growth medium supplemented with 10 µM Y27632 in a 60 mm non-adhesive plate. After 5 days, cardiac differentiation was induced by replacing the medium with RPMI/B27 + 12 µM CHIR99021 (d0). The medium was replaced with RPMI/B27 medium after 24 h (d1) and again on d2 with RPMI/B27 medium + 5 µM IWP2 + 5 µM purmorphamine (Reprocell, #04-0009) + 5 µM SB4315142 (Cayman, #13031). At d4, the medium was changed to RPMI/B27, and the cardiac spheres were cultured until they began beating (around d10), with a medium change every other day.

### Tube formation assay

Enriched PECAM1^+^ cells were seeded on a Matrigel-coated 24-well plate (250 µl Matrigel/well) with a density of 10^5^ cells/cm^2^. Images were taken after 18 h using a BZ-X800E microscope (Keyence).

### LDL uptake assay

Enriched PECAM1^+^ cells were incubated with LDL-DyLight^TM^550 (Cayman Chemical, #10011229) at a final concentration of 10 ng/ml overnight in E8 medium. Afterwards, the cells were rinsed twice with DPBS and fixed with 4% paraformaldehyde solution for 30 min.

### Immunofluorescence (IF) staining

We applied a common IF staining procedure. Briefly, cells grown on coverslips were fixed for 30 min using 4% paraformaldehyde solution, then permeabilized and blocked in DPBS containing 0.3% (v/v) Triton X-100 and 10% (w/v) bovine serum albumin (BSA; Merck, #A9418). Primary antibodies were applied in DPBS containing 10% (w/v) bovine serum albumin (BSA) at 4 °C overnight. After washing with DPBS, fixed cells were then incubated with the respective secondary antibodies in Hoechst 33342 solution (1 µg/ml) for 1 h at room temperature. Finally, the coverslips were washed again with DPBS and mounted with Aqua Poly/Mount medium (Polysciences, #18606-20). 3D spheres were first fixed with 4% paraformaldehyde solution for one hour, washed with DPBS, equilibrated in 20% (w/v) sucrose in DPBS overnight at 4 °C and frozen in TissueTek^®^ O.C.T™ Compound (Sakura Finetek™, #4583). Cryosections (8–10 μm) have been prepared and stained as described above. The antibodies used for IF are listed in Tables 1 and 2. For imaging a BZ-X800E microscope (Keyence) was used.

### Flow cytometry (FC)

Cells grown in a monolayer or 3D aggregates were dissociated with 0.05% Trypsin-EDTA to get a single cell suspension and fixed afterwards with 4% paraformaldehyde solution for 20 min at room temperature. Subsequently, they were washed twice with DPBS and then incubated in DPBS containing 0.3% (v/v) Triton X-100 and 1% (w/v) BSA for permeabilization and blocking. Incubation with primary antibody was performed in the same buffer at 4 °C overnight. After washing with DPBS, the respective secondary antibody was added for 1 h at room temperature in the dark. The antibodies used for FC are listed in Tables 1 and 2. Analysis was performed using a CyFlow Space cytometer (Sysmex) and FlowJo V10 software.

**Table 1:** Primary antibodies used for IF and FC

Primary antibodies				
	Company	Order no.	Dilution	Application
ACTA2	Merck (Sigma Aldrich)	A5228	1:800	IF
CDH5	Santa Cruz Biotechnology	Sc-9989	1:100	IF, FC
GATA4	Santa Cruz Biotechnology	Sc-25,310	1:100	IF, FC
ISL1	Abcam	ab86501	1:200	IF, FC
KDR	Abcam	ab2349	1:100	IF, FC
NFATC1	Novusbio	NB100-56732	1:100	IF, FC
NKX2.5	Santa Cruz Biotechnology	sc-14,033	1:100	IF
OCT3/4	Santa Cruz Biotechnology	Sc-9081	1:100	IF
PDGFRA	Cell Signaling Technology	3174	1:100	IF
PDGFRB	Cell Signaling Technology	3169	1:100	IF
PECAM1	Santa Cruz Biotechnology	sc-376,764	1:100	IF, FC
TAGLN	Thermo Fisher Scientific (Invitrogen)	PA5-81550	1:100	IF
TBXT	Abcam	ab209665	1:500	IF
TNNT2	Abcam	ab45932	1:100	IF

**Table 2:** Secondary antibodies used for IF and FC

Secondary antibodies				
	Company	Order no.	Dilution	Application
Alexa Fluor 488 affiniPure donkey anti-rabbit IgG	Jackson ImmunoResearch	711-545-152	1:400	IF
Alexa Fluor 647 affiniPure donkey anti-rabbit IgG	Jackson ImmunoResearch	711-605-152	1:400	IF, FC
Alexa Fluor 647 affiniPure goat anti-mouse IgG, subclass 1	Jackson ImmunoResearch	115-605-205	1:400	IF, FC
Cy3™ affiniPure donkey anti-rabbit	Jackson ImmunoResearch	711-165-152	1:400	IF
Cy3™ affiniPure goat anti-mouse IgG	Jackson ImmunoResearch	715-165-151	1:400	IF
Cy3™ affiniPure goat anti-mouse IgG, subclass 2a	Jackson ImmunoResearch	115-165-206	1:400	IF

### RNA isolation and bulk RNA-Seq analysis

Total RNA was extracted using the RNeasy Plus Micro Kit (Qiagen, #74034) and library preparation was carried out using the Revelo™ RNA-Seq High Sensitivity Kit (Tecan, # 30201359) according to the manufacturer’s instructions. 3’-mRNA sequencing was performed with NextSeq2000 (P2 flow cell, 50 bp, paired end). The Galaxy platform (Freiburg Galaxy project [13]) was used for bioinformatic analysis. The RNA sequencing reads were mapped with RNA STAR [14], reads per gene were counted by featureCounts [15], and differentially expressed genes were identified by DESeq2 [16]. Heatmap2 was used to generate heatmaps of differentially expressed genes (DEGs) with an adjusted p-value < 0.05, a log2(fold change) > 1 (log2(FC) > 1), and a z-score normalization prior to clustering. Gene ontology (GO) analysis was performed using either the Cytoscape plugin of ClueGo (pie charts) or ShinyGo (https://bioinformatics.sdstate.edu/go/, fold enrichment scores). Gene set enrichment analysis was performed using the GSEA 4.4.0 software (Broad Institute, https://www.gsea-msigdb.org/gsea/msigdb/index.jsp). For the calculation of the transcripts per million (TPM), first, the number of reads for each gene was divided by the gene length in kb to obtain the reads per kilobase (RPK). This value was then divided by a scaling factor (= sum of all RPK per gene divided by 10^6^) to calculate the TPM values.

### Statistical analysis

Data are presented as mean ± SEM. Student’s t-test or one-way analysis of variance (ANOVA) with Tukey’s post-hoc test was performed with GraphPad Prism 10. Differences were considered statistically significant when *p* < 0.05.

## Results

### VELCs can be derived from hiPSCs in 2D

As valve endocardial cells originate from multipotent cardiovascular progenitor cells (CPCs) [17], we first tested their derivation from human induced pluripotent stem cells (hiPSCs) using a published protocol [7]. Here, hiPSCs were differentiated into CPCs by sequential treatment with Wnt3a/BMP4 and FGF2/BMP4. Subsequently, CPCs were reported to further differentiate into valve endothelial-like cells (VELCs) by application of BMP4, TGFβ1, and VEGF. The transdifferentiation of VELCs into valve interstitial-like cells (VILCs) occurred through induction of endothelial-to-mesenchymal transition (EndMT) [18] upon application of FGF2 (100 ng/ml) and TGFβ1 (50 ng/ml) for 6 days (Suppl. Figure 1 A). However, when using this protocol, we observed that although differentiation into VELCs was efficient, they were not stable in culture showing signs of a spontaneous (trans)differentiation between day 10 (d10) and day 16 (d16), namely: (i) The cells underwent clear morphological changes becoming more elongated and less cobblestone-shaped (Suppl. Figure 1E, I). (ii) Bulk RNA-Seq data showed an increased expression of mesenchymal genes such as *COL1A1*, *POSTN*, *VIM*,* CDH2*, and *TWIST1*, along with a decline in some epithelial genes (Suppl. Figure 1B). Gene set enrichment analysis (GSEA) confirmed enrichment of genes associated with epithelial-to-mesenchymal transition (a predefined gene set for EndMT was not available) (Suppl. Figure 1C). (iii) Principal component analysis (PCA) showed that d16 VELCs were more similar to d16 VILCs after EndMT induction than to d10 VELCs (Suppl. Figure 1D). These findings were further corroborated by immunostaining for various cell type markers. At d10, most cells were PECAM1^+^ and NFATC1^+^, confirming their endocardial identity, and only a few cells expressed ACTA2 as a mesenchymal marker (Suppl. Figure 1F-H). However, between d10 and d16, most cells became PECAM1^−^ and NFATC1^−^, while an increase in ACTA2^+^ cells was observed (Suppl. Figure 1J-L), suggesting spontaneous transdifferentiation into a mesenchymal phenotype.

We therefore modified the protocol [7] to produce a stable VELC population. The initial step of differentiating CPCs remained unchanged, but for in vitro differentiation of VELCs, we excluded BMP4 and TGFβ1, which have both been reported to induce EndMT [19] and thereby might affect the stability of differentiating VELCs. Instead, we used VEGF as the only factor (Fig. 1A) to guide CPCs into VELCs and prevent their transdifferentiation into mesenchymal cells (Fig. 1A). The characterization of CPCs at d4 revealed that, as expected, most cells expressed characteristic cardiac progenitor markers like GATA4 and ISL1 in the nucleus and KDR on the cell membrane (Fig. 1B–D). Flow cytometry data confirmed that a high proportion of cells expressed these markers, with 80.38 ± 4.74% of the cells being GATA4^+^, 78.28 ± 3.25% ISL1^+^, and 73.05 ± 4.27% KDR^+^ (Fig. 1E–H).

Further differentiation into VELCs was achieved, as mentioned above, by treating CPCs with VEGF (100 ng/ml) for 6 days. In immunofluorescence staining, the differentiated VELCs expressed the cardiac GATA4 and the endocardial NFATC1 (located in the nucleus) markers, as well as the endothelial markers PECAM1 and CDH5, confirming their phenotype (Fig. 1I–L). This was further supported by flow cytometry analysis, which showed that VEGF treatment resulted in 85.72 ± 2.52% GATA4^+^, 87.2 ± 1.76% PECAM1^+^, and 89.96 ± 2.32% NFATC1^+^ cells at day 10 (Fig. 1P). Therefore, using VEGF alone, instead of combining it with other factors, effectively generated hiPSC-derived VELCs in 2D.

### Use of VEGF as main driver increases the stability of hiPSC-derived 2D VELCs

**Fig. 2:**
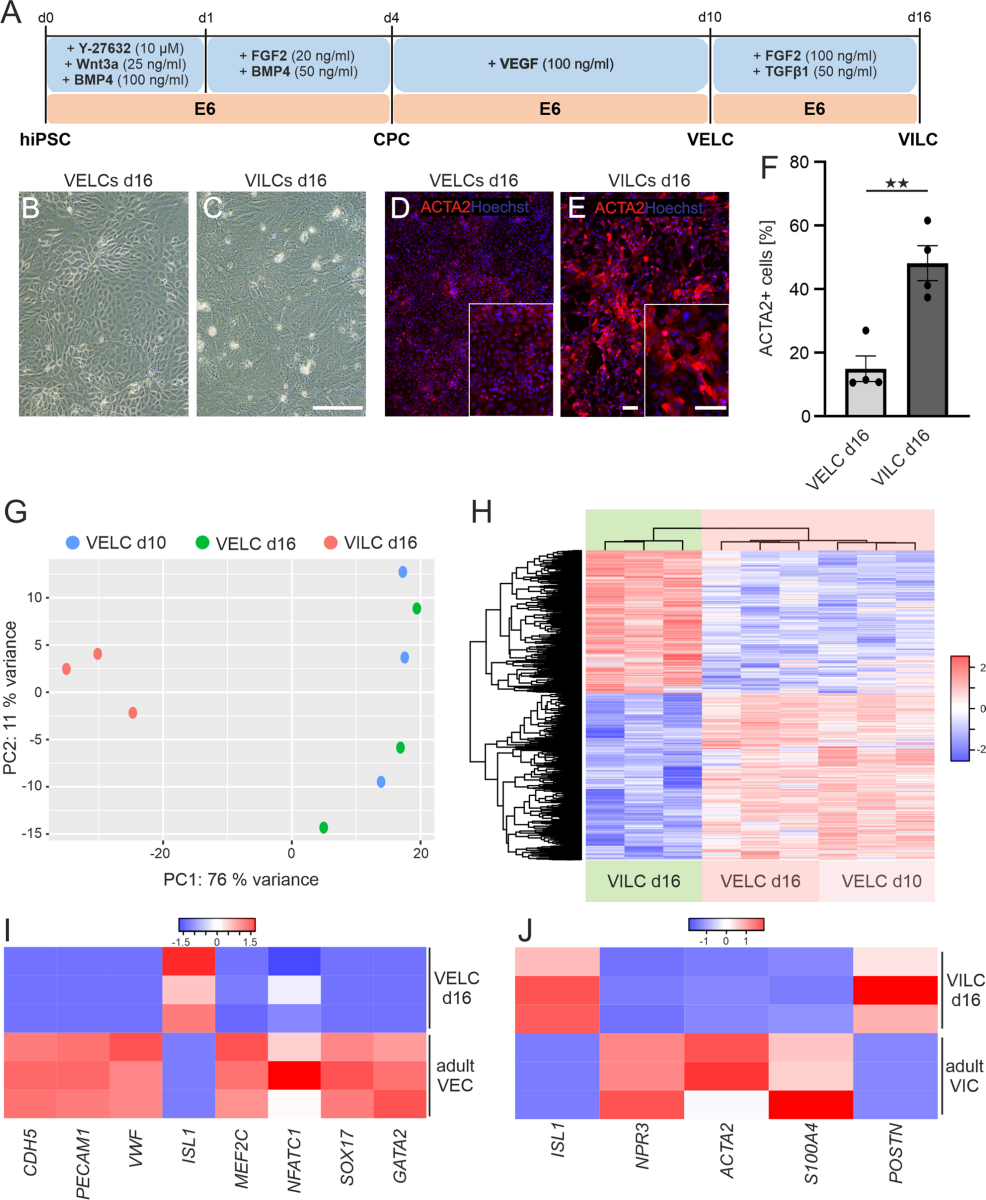
Bulk RNA-Seq analysis of hiPSC-derived VELCs and VILCs and adult VECs and VICs. **A** Schematic differentiation protocol for hiPSC-derived VELCs and their transdifferentiation into VILCs by induction of EndMT. **B**, **C** Phase contrast images of VELCs after treatment with 100 ng/ml VEGF (**B**) or with 100 ng/ml FGF2 and 50 ng/ml TGFβ1 for 6 days (**C**); scale bar = 100 μm. **D**, **E** Immunofluorescence staining of VELCs after treatment with 100 ng/ml VEGF (**D**) or with 100 ng/ml FGF2 and 50 ng/ml TGFβ1 for 6 days (**E**); scale bar = 100 μm. **F** Quantification of the percentage of ACTA2^+^ cells in controls (VELC d16) and after EndMT induction (VILC d16) (*n* = 4). **G** Principal component analysis (PCA) based on bulk RNA-Seq data for hiPSC-derived VELCs and VILCs using the 2D differentiation protocol shown in A (*n* = 3). VELCs at day 10 (d10) were either cultivated in the presence of VEGF for 6 days (VELC d16) or transdifferentiated into VILCs by application of FGF2 and TGFβ1 for 6 days (VILC d16). **H** Heatmap showing the most differentially expressed genes (DEGs) in VELCs at d10 and d16, and VILCs at d16 (*n* = 3). **I** Heatmap comparing the expression of endothelial and endocardial markers in VELCs at d16 with adult VECs (*n* = 3). **J** Heatmap comparing the expression of endocardial and interstitial markers in VILCs at d16 with adult VICs (*n* = 3). Data are shown as mean ± SEM. Statistical analysis was performed using Student’s t-test; * *p* ≤ 0.05, ** *p* ≤ 0.01, *** *p* ≤ 0.001, **** *p* ≤ 0.0001. For heatmaps only differentially expressed genes with a log2(FC) > 1 and an adj. p-value < 0.05 have been considered

As the VELCs derived with the published protocol were found to be unstable in cell culture, we first verified this aspect in VELCs differentiated with our modified protocol. In addition, we assessed their ability to undergo EndMT and to transdifferentiate into VILCs upon EndMT induction with FGF2 (100 ng/ml) and TGFβ1 (50 ng/ml) (Fig. 2A). Six days after treatment with FGF2 and TGFβ1 (Fig. 2A, VILC d16) or maintenance in the presence of VEGF ( VELC d16), we observed typical morphological changes in VELCs exposed to FGF2 and TGFβ1, with most cells losing their cobblestone-like pattern and adopting a more spacious and elongated cell shape (Fig. 2B and C). Furthermore, immunostaining proved that the number of ACTA2^+^ cells, a marker of more mature VILCs, increased approximately threefold from 14.92 ± 4.02% (VELC d16) to 48.12 ± 5.5% (VILC d16) following EndMT induction (Fig. 2D–F), indicating robust transdifferentiation.

To further examine the stability and identity of the cells obtained with the new differentiation protocol, we performed bulk RNA-Seq analysis. The PCA plot of VELCs at d10 and d16 and VILCs at d16 showed that the variation between VELCs at the two different time points was low, but significantly different from the VILCs (Fig. 2G). This was further underscored by the heatmap showing the most differentially expressed genes (DEGs), which revealed that d16 and d10 VELCs cluster together, confirming their stability over time, whereas VILCs exhibited an entirely different expression profile (Fig. 2H). We also investigated the molecular features and maturation levels of hiPSC-derived VELCs and VILCs compared to adult human valve endothelial (VECs) and interstitial cells (VICs) using bulk RNA-Seq analysis. Analysis of general endocardial markers, such as *ISL1*,* MEF2C*,* NFATC1*, *SOX17*, as well as hematopoietic/endothelial markers for VELCs (*GATA2*, *CDH5*,* PECAM1*,* VWF*) or mesenchymal markers for VILCs (*ACTA2*,* S100A4*,* POSTN*) revealed, as expected, differences between the hiPSC-derived cells and their adult counterparts (Fig. 2I, J): hiPSC-derived VELCs expressed lower levels of all three analyzed endothelial markers compared to adult VECs (Fig. 2I). Similarly, the expression of mesenchymal markers was lower in VILCs compared to adult VICs (Fig. 2J), indicating also here an incomplete maturation of the differentiated cells. To compare hiPSC-derived VELCs with adult VECs, an additional analysis using a broader gene panel, previously established [7] to discriminate between endocardial cushion cells (early-stage endocardial cells) and embryonic VECs (late-stage endocardial cells), was performed (Suppl. Figure 2). This gene set includes general endothelial markers (e.g., *CDH5*,* PECAM1*,* VWF*,* KDR*,* EMCN*), proposed early-stage marker genes such as *GATA4*,* SOX17*,* JAG1*, and *NOTCH3*, and potential late-stage marker genes such as *HEY2*,* HAND2*,* SMAD6*, and *CAV1*. Here, all endothelial markers were upregulated in adult VECs, as were most of the markers from the late-stage marker gene panel, whereas higher expression of early-stage marker genes was observed in VELCs (Suppl. Figure 2). Thus, we established a 2D differentiation protocol using VEGF alone to produce stable VELCs from CPCs and found that these VELCs could be efficiently converted into VILCs after treatment with FGF2 and TGFβ1.

### 3D cardiac progenitor spheres express GATA4, ISL1, KDR and PDGFRA

**Fig. 3:**
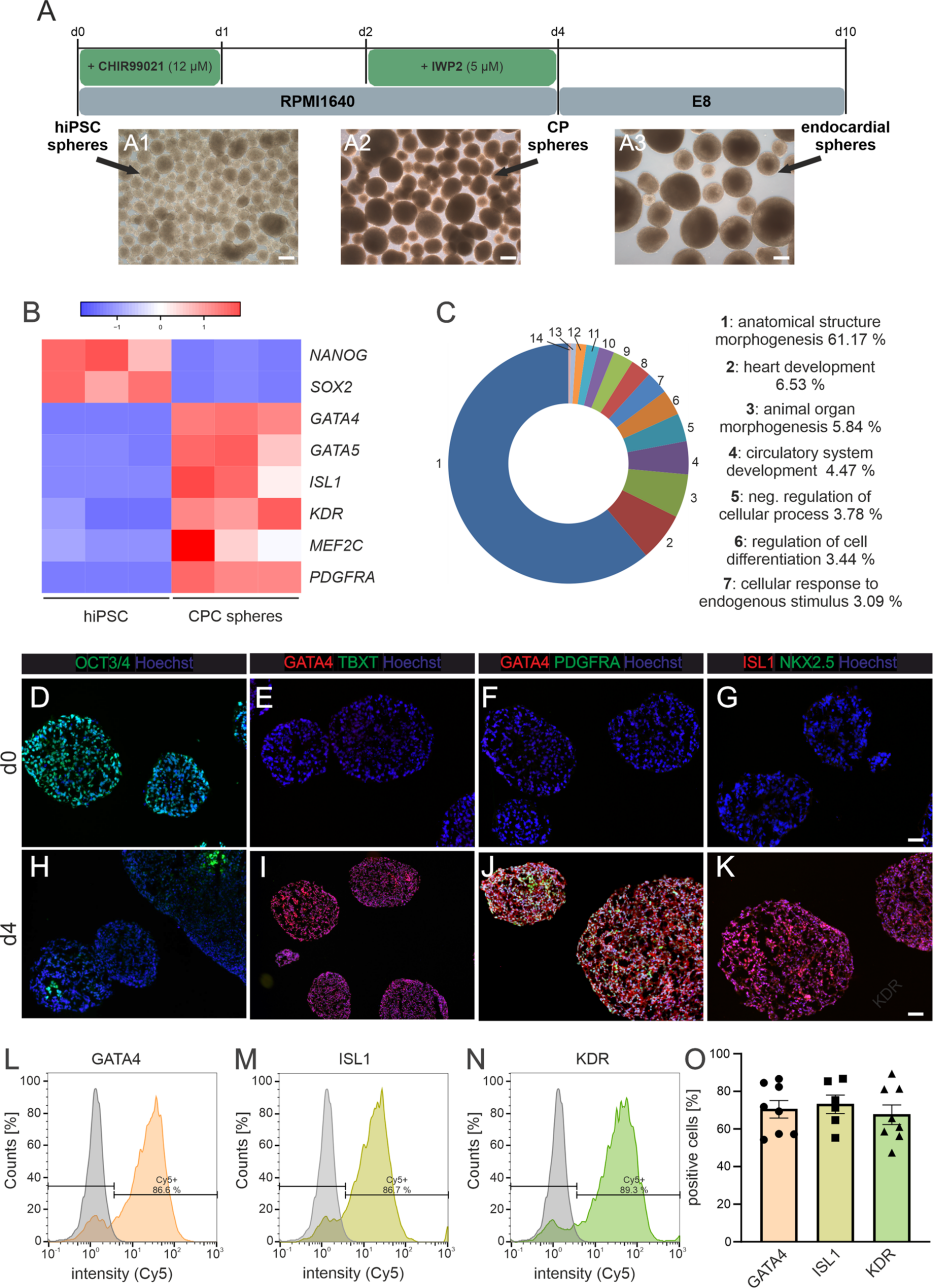
3D differentiation protocol for the generation of endocardial spheres. **A** First, hiPSC spheres (A1) were differentiated into cardiac progenitor (CP) spheres (A2) by applying 12 µM CHIR99021 for 24 h, followed by a 24 h rest, and subsequent treatment with 5 µM IWP2 for 48 h. Further differentiation of CP spheres into endocardial spheres (A3) was performed by switching the cultivation medium from RPMI1640 to E8 medium at day 4 (d4); scale bar = 200 µM. **B** Heatmap showing expression levels of pluripotency and cardiac progenitor markers in CP spheres at d4 compared to undifferentiated hiPSCs at d0 (*n* = 3). **C** Gene ontology (GO) analysis of upregulated genes related to biological processes in CP spheres at d4 compared to undifferentiated hiPSCs at d0; 8: supramolecular fiber organization 2,75%, 9: morphogenesis of an epithelium 2.75%, 10: cell morphogenesis 2.06%, 11: urogenital system development 1.72%, 12: striated muscle tissue development 1.37%, 13: response to wounding 0.69%, 14: ECM organization 0.34%. **D**–**G** Immunofluorescence staining of hiPSC spheres at d0; scale bar = 50 µM. **H**–**K** Immunofluorescence staining of CP spheres at d4; scale bar = 50 μm. **L**–**O** Flow cytometry histograms (**L**–**N**) and quantification (**O**) for GATA4, ISL1, and KDR expression (single stainings) in CP spheres at d4 (*n* ≥ 6). For heatmaps only differentially expressed genes with a log2(FC) > 1 and an adj. p-value < 0.05 have been considered

Three-dimensional (3D) suspension cultures are known to create a more physiological environment, promoting better cell-cell interaction and self-organization. We therefore aimed to develop a 3D suspension protocol for VELC generation. Since the key step in achieving VELC development is the differentiation into CPCs, we probed the small-molecule-based GiWi (GSK inhibition/Wnt inhibition) protocol, which is known to generate hiPSCs-derived cardiomyocytes with CPCs as an intermediate stage [12, 20], to differentiate hiPSC into cardiac progenitor (CP) spheres (Fig. 3A). To this end, we aggregated 10^6^ hiPSCs in 5 ml medium per 6cm dish into 3D hiPSC spheres, cultivated them in suspension culture for 4–5 days (Fig. 3A1), and treated them with CHIR99021, which promotes hiPSC differentiation toward a meso-endodermal fate.

This was followed by the addition of the Wnt inhibitor IWP2, which promotes cardiac lineage induction [20] to generate CP spheres until d4 of differentiation (Fig. 3A2). Then, these were further differentiated into endocardial spheres until d10 (Fig. 3A3). We observed that the spheres continuously grew in size during the differentiation process. They increased from a diameter of 185.7 ± 11.54 μm in hiPSC spheres (*n* = 33) to 281.4 ± 18.84 μm in CP spheres (*n* = 28), and to 437.7 ± 40.94 μm in endocardial spheres (*n* = 17), but did not exhibit obvious morphological changes.

We characterized the cells at the different time points using bulk RNA-Seq and found they efficiently differentiated into CPCs. When comparing the expression of pluripotency markers (*NANOG*,* SOX2*) and CPC markers (*GATA4*,* GATA5*,* ISL1*,* KDR*,* MEF2C*,* PDGFRA)* in hiPSC and CP spheres using a heatmap, it was observed that during differentiation the CPC markers were clearly upregulated in the latter (Fig. 3B). Gene ontology (GO) analysis of biological processes revealed that the significantly upregulated genes (CP spheres vs. hiPSC spheres) belong to terms related to anatomical structure morphogenesis (61.17%), heart development (6.53%), animal organ morphogenesis (5.84%) and circulatory system development (4.47%), as expected for processes linked to organ development with a focus on the heart (Fig. 3C). An additional enrichment plot confirmed these results, showing that the calculated fold enrichment score was highest for the GO terms heart development, circulatory system development, animal organ morphogenesis, tube morphogenesis, and tube development (Suppl. Figure 3).

We next characterized the 3D structures in detail by performing immunofluorescence staining of cryosections of hiPSCs (d0) and CP spheres (d4). These results were in accordance with the gene expression data, showing a decrease in the pluripotency marker OCT3/4 (Fig. 3D, H). Interestingly, we detected no expression of the mesodermal marker TBXT (Fig. 3E, I), but there was increased expression of cardiac-lineage commitment markers such as GATA4 (Fig. 3F, J) and ISL1 (Fig. 3G, K) in CP spheres, indicating the successful differentiation of hiPSCs into CPCs. In addition, within CP spheres, a population of GATA4^+^/PDGFRA^+^ cells was observed (Fig. 3J), confirming their cardiac progenitor state, as GATA4 is activated early in cardiac specification and PDGFRA is known as a marker of multipotent cardiovascular progenitor cells [21, 22]. Since we were unable to detect NKX2.5 expression in immunofluorescence stainings of the CP spheres (Fig. 3G, K), we considered an NKX2.5^low^/ISL1^high^ CPC population, which was also consistent with observations at later time points of differentiation (Suppl. Figure 4). To further quantify CP marker expression, we performed flow cytometry and found 70.45 ± 4.65% GATA4, 73.13 ± 4.94% ISL1^+^ and 67.6 ± 5.2% KDR^+^ ells (Fig. 3L–O), which aligns with our immunocytochemistry results and confirms the CP state of the spheres at d4.

### FGF2 drives the differentiation of endocardial spheres in 3D

Next, we studied how to differentiate CP spheres into endocardial spheres and started optimizing the differentiation medium. Based on our 2D growth factor-driven protocol, we selected E6 medium as the basal medium and tested different concentrations of FGF2, since it was reported that FGF2 is sufficient to differentiate endocardial cells [6]. CP spheres grown in E6 medium for 6 days (without FGF2) showed different cell types in immunofluorescence staining, including TNNT2^+^ cardiomyocytes, ACTA2^+^ smooth muscle cells, and CDH5^+^ endothelial/endocardial cells (Suppl. Figure 5 A-D). On one hand, this reflected the multipotential differentiation ability of CP spheres, but on the other hand, it highlighted that basal E6 medium is not sufficient to differentiate endocardial cells to high levels. Using E6 medium supplemented with 50 ng/ml FGF2 or E8 medium, which already contains 100 ng/ml FGF2 [23], we noticed a decrease in the number of cardiomyocytes and stromal cells in immunofluorescence staining of endocardial spheres depending on the FGF2 concentration (Suppl. Figure 5E-L). In addition, the percentage of CDH5^+^ cells, which are expected to be endocardial cells, was low in spheres differentiated in basal E6 medium, but increased when E6 medium was supplemented with 50 ng/ml FGF2, reaching its highest levels in spheres differentiated in E8 medium (Suppl. Figure 5I-L). Based on these results, we chose the E8 medium for all subsequent differentiations of endocardial spheres to ensure low levels of cardiomyocytes and stromal cells and high levels of endocardial cells.

### 3D endocardial spheres express GATA4, PECAM1 and NFATC1

**Fig. 4:**
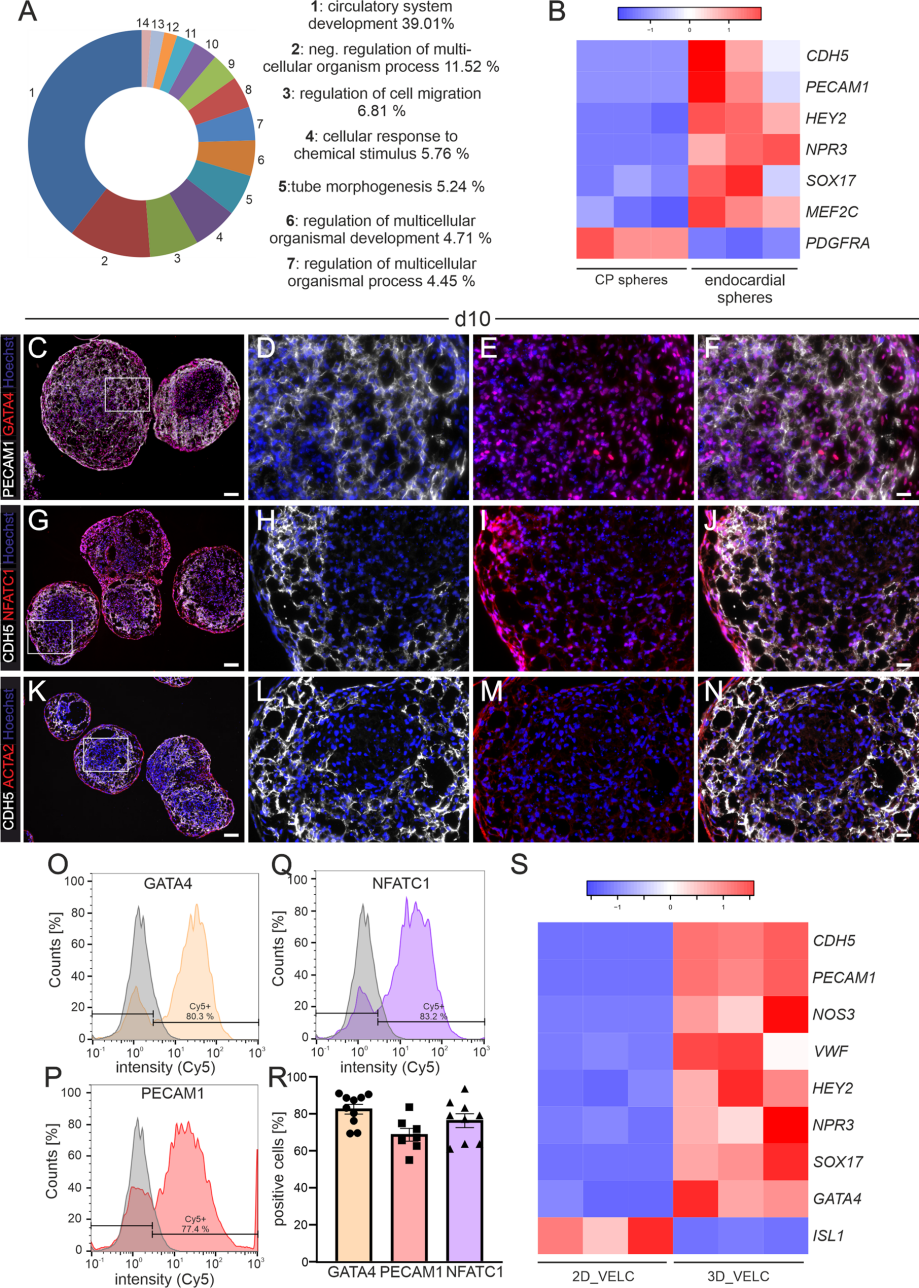
Characterization of endocardial spheres at day 10 of differentiation. **A** Gene ontology (GO) analysis of upregulated genes related to biological processes in endocardial spheres at d10 compared to CP spheres at d4; 8: blood vessel development 4.19%, 9: regulation of transport 3.93%, 10: cell migration 3.4%, 11: regulation of signaling 2.62%, 12: regulation of localization 1.83%, 13: inflammatory response 1.83%, 14: positive regulation of locomotion 1.31%, 15: circulatory system process 1.31%, 16: homeostatic process 1.31%, 17: actin cytoskeleton organization 0.79%. **B** Heatmap showing expression levels of cardiac progenitor and endocardial markers in endocardial spheres at d10 compared to CP spheres at d4. **C**–**N** Immunofluorescence staining of endocardial spheres at d10. (C, G,K) Overview images, scale bar = 100 μm. **D**–**F**, **H**–**J**, **L**–**N** Close-up images; scale bar = 20 μm. **O**–**R** Flow cytometry histograms (**O**–**Q**) and quantification (**R**) for GATA4, PECAM1, and NFATC1 expression (single stainings) in endocardial spheres at d10 (*n* ≥ 7). **S** Heatmap showing expression levels of endothelial and endocardial markers in VELCs at d16 derived from either the 2D or the 3D differentiation protocol (*n* = 3). Data are shown as mean ± SEM. Statistical analysis was performed using one-way ANOVA; * *p* ≤ 0.05, ** *p* ≤ 0.01, *** *p* ≤ 0.001, **** *p* ≤ 0.0001. For heatmaps only differentially expressed genes with a log2(FC) > 1 and an adj. p-value < 0.05 have been considered

We further examined the molecular features of the endocardial spheres in greater detail by performing additional bulk RNA-Seq data analyses comparing endocardial and CP spheres. Analysis of GO terms revealed that the majority of the upregulated genes (endocardial spheres vs. CP spheres) are associated with GO terms such as circulatory system development (38.14%) and negative regulation of multicellular organism process (11.34%) (Fig. 4A). These findings suggest a shift from general structure morphogenesis and heart development, typical of CP spheres, toward heart and vasculature development, consistent with endocardial cell differentiation. In addition, a heatmap comparing the expression of cardiac progenitor markers (*MEF2C*,* PDGFRA*) and endocardial markers (*CDH5*,* PECAM1*,* HEY2*,* NPR3*,* SOX17*), showed an upregulation of *MEF2C* and endocardial markers in endocardial spheres. Meanwhile, the expression of *PDGFRA*, a marker for multipotent CPCs, was higher in CP spheres (Fig. 4B), confirming the transition of CP into endocardial spheres.

We also analyzed the cellular composition of endocardial spheres on day 10 using immunofluorescence staining. This revealed PECAM1^+^/GATA4^+^ cells (Fig. 4C–F) and CDH5^+^/NFATC1^+^ cells (Fig. 4G–J), which were mostly negative for ACTA2 as an interstitial marker (Fig. 4K–N), highlighting the endocardial identity of the spheres. To further underscore this result, we used flow cytometry. The findings demonstrated high efficiency of VELC generation in our 3D differentiation protocol, as 82.51 ± 2.62% of the cells were GATA4^+^, 68.69 ± 3.5% were PECAM1^+^, and 76.27 ± 3.74% expressed NFATC1, indicating high percentages of cells expressing markers typical for VELCs (Fig. 4O–R).

Next, we examined the maturation of the VELCs and addressed this via bulk RNA-Seq analysis. Interestingly, we found that VELCs derived from the 3D differentiation protocol exhibited increased expression of the endothelial markers *PECAM1*,* VWF*, and *NOS3*, as well as the endocardial markers *HAND2*,* HEY2*,* NFATC1*, and *GATA4*, compared to VELCs derived from the 2D protocol described above (Fig. 4S), suggesting higher maturity of VELCs generated in 3D spheres. In contrast, *ISL1* was found to be stronger expressed in 2D VELCs, indicating a premature progenitor phenotype of VELCs derived from 2D differentiation (Fig. 4S). This was further confirmed by a direct comparison of 2D VELCs and 3D VELCs with adult VECs using a broader gene panel, established by Cheng et al. [7] (Suppl. Figure 6A). The heatmap revealed higher expression of genes belonging to the endothelial and late endocardial marker panel in adult VECs, whereas 2D-derived VELCs mainly showed increased expression of genes assigned to the early endocardial marker panel (Suppl. Figure 6A). 3D VELCs, which expressed endothelial and some late endocardial marker genes but hardly any early endocardial marker genes, can be considered more mature than 2D VELCs but less mature than adult VECs (Suppl. Figure 6A). To provide an additional direct comparison of 3D VELCs and adult VECs, we calculated transcripts per million (TPMs) for each in a distinct panel of either general endothelial markers (*CDH5*,* PECAM1*,* TEK*,* FLT1*,* KDR*,* ICAM2*,* NOS3*,* VWF*, Suppl. Figure 6B) or endocardial markers (*GATA4*,* HEY2*,* MEF2C*,* ISL1*,* NPR3*,* NFATC1*, Suppl. Figure 6C). All of the previously mentioned markers are expressed in 3D VELCs as well as in adult VECs, confirming the endocardial character of VELCs and indicating a certain degree of similarity between these two cell populations. Significant differences were observed for *PECAM1*,* FLT1*,* KDR*, and *MEF2C* (higher TPMs in 3D VELCs) as well as for *NOS3* and *VWF (*higher TPMs in adult VECs), suggesting that adult VECs are more mature. Importantly, we also tested our new 3D in vitro differentiation protocol for VELCs with a different hiPSC line and successfully reproduced the generation of CDH5^+^/NFATC1^+^ spheres, confirming the validity of our protocol (Suppl. Figure 7A-D).

### 3D-derived VELCs are able to undergo EndMT and transdifferentiate into VILCs

**Fig. 5:**
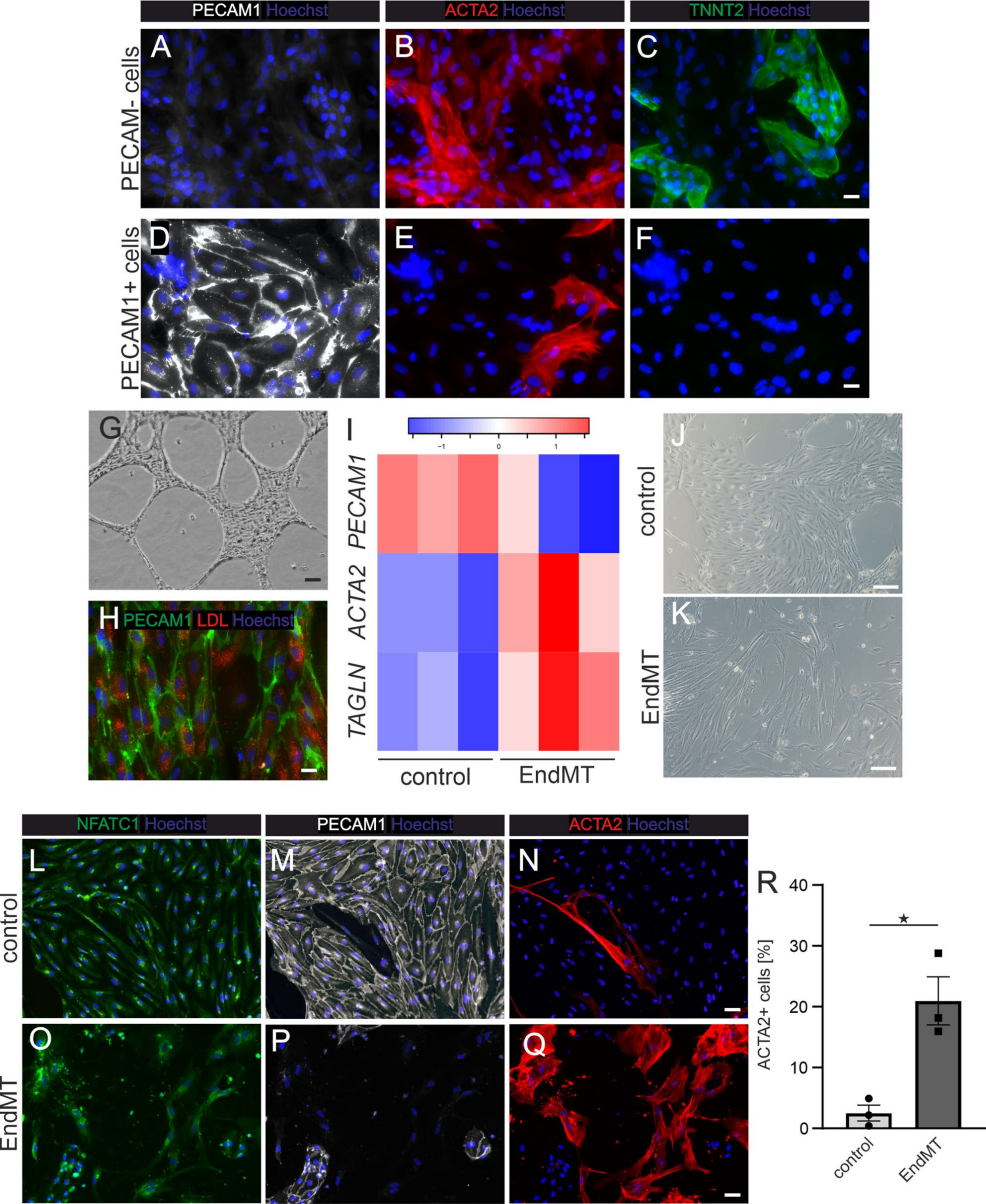
Characterization of VELCs derived from endocardial spheres. **A**–**F** Immunofluorescence staining of dissociated endocardial spheres, which have been sorted into PECAM1^−^ cells (**A**–**C**) and PECAM1^+^ cells (**D**–**F**) via MACS; scale bar overview = 100 μm, scale bar close-up = 20 μm. **G** Network formation of PECAM1^+^ cells derived from endocardial spheres in Matrigel; scale bar = 100 μm. **H** LDL uptake of PECAM1^+^ cells derived from endocardial spheres; scale bar = 20 μm. **I** Heatmap of EndMT marker for VELCs and VILCs derived from endocardial spheres at day 16 (*n* = 3). **J** and **K** Phase contrast images of PECAM1^+^ cells derived from endocardial spheres without (**J**) or with (**K**) treatment with 200 ng/ml TGFβ1 for EndMT induction. **L**–**Q** Immunofluorescence staining of PECAM1^+^ cells derived from endocardial spheres without (**L**–**M**) or with (**O**–**Q**) treatment with 200 ng/ml TGFβ1 for EndMT induction; scale bar = 50 μm. **R** Quantification of ACTA2^+^ cells after EndMT induction with 200 ng/ml TGFβ1 (*n* = 3). Data are shown as mean ± SEM. Statistical analysis was performed using Student‘s t-test; * *p* ≤ 0.05, ** *p* ≤ 0.01, *** *p* ≤ 0.001, **** *p* ≤ 0.0001. For heatmaps only differentially expressed genes with a log2(FC) > 1 and an adj. p-value < 0.05 have been considered

As pointed out above, EndMT is a critical step for valve development in the embryo and for triggering AVS in adults. Therefore, we proved the ability of VELCs differentiated within 3D endocardial spheres to undergo EndMT and transdifferentiate into VILCs. To this end, the endocardial spheres were treated for 6 days with 200 ng/ml TGFβ1. Using immunofluorescence staining, we investigated the transition of CDH5^+^/ACTA2^−^/TAGLN^−^ VELCs to CDH5^−^/ACTA2^+^/TAGLN^+^ VILCs. Compared to the control group (Suppl. Figure 8A-D), we observed an increase in TAGLN^+^ cells after EndMT induction, but no significant decrease in CDH5^+^ cells (Suppl. Figure 8E-H), indicating a very low EndMT efficiency under these conditions. We suspected that this might be due to specific local conditions within the spheres and/or inadequate penetration of the EndMT-inducing growth factor. To bypass this, we dissociated the endocardial spheres at d10 using a cardiomyocyte dissociation kit, enriched the VELC population by magnet-assisted cell sorting (MACS) for PECAM1, with the goal of inducing EndMT in plated 3D-derived VELCs. Prior to EndMT induction, we characterized the enriched VELCs using immunofluorescence staining and functional assays. After sorting, the cells of the flowthrough appeared PECAM1^−^ in immunofluorescence staining, but expressed ACTA2 and TNNT2 (Fig. 5A–C).

In contrast, the magnetically labeled and retained cells mainly expressed PECAM1 (Fig. 5D–F), confirming the successful enrichment and plating of PECAM1^+^ VELCs derived from 3D endocardial spheres. The ability of these cells to form networks in Matrigel and to uptake LDL further reinforced their endocardial character (Fig. 5G, H).

To induce EndMT in these enriched VELCs, we applied 200 ng/ml of TGFβ1 for 6 days. Analysis of bulk RNA-Seq data indicated successful EndMT induction, as the expression of the endothelial marker *PECAM1* was downregulated after treatment with TGFβ1, while *ACTA2* and *TAGLN* were upregulated (Fig. 5I). Accordingly, typical morphological changes associated with EndMT were observed under the microscope, as the cells adopted the characteristic elongated phenotype and developed more cell protrusions (Fig. 5J, K). This was accompanied by a shift in NFATC1 expression from the nucleus to the cytoplasm (Fig. 5L, O), a loss of the characteristic PECAM1 expression (Fig. 5M, P), and an increase in ACTA2^+^ cells (Fig. 5N, Q) from 2.51 ± 1.3% to 20.96 ± 3.96% after EndMT induction (Fig. 5R). So, we successfully developed a simple and effective 3D protocol to generate endocardial spheres in a physiological microenvironment. Furthermore, these spheres serve as a source of VELCs, which can be transdifferentiated into VILCs upon EndMT induction.

## Discussion

Given the clinical importance of acquired and genetic forms of AVS combined with the current lack of mechanistic insights into disease mechanisms, biomarkers, and curative treatments, novel human in vitro models are urgently needed.

Therefore, in recent years, differentiation protocols for generating hiPSC-derived endocardial cells, like valve endothelial-like cells (VELCs), have been published, providing tools to study development and experimental treatment options for AVS in vitro. All of them use 2D monolayer differentiation methods, except Mikryukov et al., who developed a 3D protocol limited to the mesodermal stage, covering only the first three days of differentiation [5, 7–9]. A common feature of all these protocols is the induction of either cardiac mesoderm or cardiac progenitor cells (CPCs) in the first step. For the subsequent differentiation step, which involves generating endocardial cells like VELCs, two main strategies can be identified: either applying the combination of FGF2 and BMP10 [6, 8] or, alternatively, using a high dose of VEGF combined with BMP4 and TGFβ1 [7] or forskolin [9].

Since CPCs originate from the lateral plate mesoderm [24, 25], they are committed to the cardiac lineage and well-suited to differentiate into VELCs. Therefore, we selected a straightforward protocol in which Wnt3a, BMP4, and FGF2 are used to generate CPCs, followed by a growth factor cocktail for differentiating into VELCs [7]. Although CPC generation proved efficient, we observed that the resulting VELC population was unstable and underwent spontaneous transdifferentiation into a mesenchymal fate under the reported growth factor cocktail. We suspected that BMP4 and TGFβ1 could be responsible for this, since their contributions to EndMT induction have been reported previously [19]. We therefore tested the application of a high dose of VEGF, which is believed to prevent EndMT and thereby stop the spontaneous transdifferentiation of VELCs [26], to differentiate the CPC population into stable VELCs. This change produced GATA4^+^/PECAM^+^/NFATC1^+^ VELCs, that remained stable over time without the previously observed spontaneous transdifferentiation into mesenchymal cells. Furthermore, these VELCs could be effectively induced to undergo complete EndMT (≈ 48% ACTA2^+^ cells), which is a relatively high percentage when compared to Mikryukov et al., who reported about 30% of VILCs after EnMT induction, and Liu et al., who reported a range of 10–73% of transdifferentation depending on the hiPSC lines used [6, 8]. One potential future approach to further improve the stability of VELCs and increase the efficiency of EndMT and VILC generation in 2D could be an extra sorting step (e.g. MACS) to significantly enrich the VELC population. Recently, Cai et al. published a similar differentiation protocol for hiPSC-derived VELCs [9]. However, unlike our 2D protocol, CHIR and BMP4 were used to differentiate hiPSCs into cardiac lateral plate mesoderm (CLPM) in the first step, followed by a combination of VEGF and forskolin in the second step to generate VELCs. Interestingly, the intermediate-stage CLPM expressed ISL1 and NKX2.5, with higher levels of NKX2.5 [9], whereas the CPCs generated here were reported as ISL^+^/NKX2.5^low^ CPCs [7], indicating the formation of different progenitor subpopulations as the first step toward further endocardial specification in both protocols. When assessing the efficiency of differentiated CDH5^+^/PECAM1^+^ VELCs, Cai et al. achieved 66.3 ± 1.91%, while we obtained 87.2 ± 1.76% demonstrating that the generatin of VELCs from ISL1^+^/NKX2.5^low^ CPCs is substantially more effective. Furthermore, Cai et al. reported the differentiation of CDH5^+^ VELCs and CDH5^−^ VILCs in parallel without inducing EndMT to transdifferentiate VELCs into VILCs [9]. In contrast, our 2D differentiation protocol produced a stable VELC population where EndMT can be induced to generate VILCs, which is beneficial for studying developmental aspects of cardiac valve formation.

Given the clear benefit of a more organotypic environment, which has already been shown to enhance the maturity of other cell types such as cardiomyocytes [27], hepatocytes [28], or neurons [29], and offers the potential for upscaling [30], we next established and evaluated a 3D differentiation protocol for hiPSC-derived VELCs. Based on our results from VELC differentiation in 2D, we planned to use CPCs as an intermediate stage in 3D as well. Therefore, we tested the potential of the small-molecule-based GiWi (GSK inhibition/Wnt inhibition) approach [12, 20], which is a well-established protocol for differentiating hiPSCs into cardiomyocytes, to generate intermediate CPCs within our 3D differentiation protocol. Our results indicate that the GATA4^+^/ISL1^+^ CPCs derived from the GiWi protocol in 3D conditions are multipotent, as they co-express PDGFRA [21]. This serves as a marker for progenitor cells that primarily contribute to the vascular and mesenchymal components of the developing human heart. These cells can differentiate not only into cardiomyocytes, but also into ACTA2^+^ smooth muscle cells, myofibroblasts, and PECAM1^+^/NFATC1^+^ VELCs [21]. Besides the characteristic CPC markers GATA4 and ISL1, NKX2.5 is often used as a marker for endocardial lineage differentiation. However, existing publications are somewhat contradictory, as Mikryukov et al. used NKX2.5 to distinguish between endocardial and endothelial cells, while Cheng et al. utilized ISL1^+^KDR^+^NKX2.5^low^ CPCs to differentiate endocardial VELCs [6, 7]. Our data support the latter, as hiPSC-derived CP spheres were ISL1^+^/NKX2.5^low^ and, more importantly, turned out to be NFATC1^+^, which is the most specific developmental marker for the endocardial lineage [31]. Further differentiation of CP spheres into GATA4^+^/PECAM1^+^/CDH5^+^/NFATC1^+^ endocardial spheres was achieved using E8 medium, which contains 100 ng/ml FGF2 in its basal composition [23], indicating that applying this growth factor alone is sufficient for endocardial specification of CPCs in 3D. This matches the results from Mikryukow et al., who used FGF2 in combination with BMP10 to differentiate hiPSC-derived VELCs, but also showed that FGF2 alone induces endocardial differentiation [6].

A key characteristic of VELCs during development and in disease is their ability to undergo EndMT and transdifferentiate into VILCs. Different pathways such as TGFβ, BMP, or Wnt/β-catenin are involved in this process during heart development and coordinate the transition of some – but not all – VELCs into VILCs, suggesting a high level of complexity [32]. Based on the well-characterized induction of EndMT in valve endothelial cells through the TGFβ pathway [18, 33], we used a high dose of 200 ng /ml TGFβ1 to induce EndMT in 3D endocardial spheres, but it proved to be insufficient. Here, alternative strategies could be tested, including using only small spheres, reaggregating PECAM1^+^ cells after dissociation of endocardial spheres, or using growth factors such as combining TGFβ1 with IL1β, using TGFβ2 instead of TGFβ1, or applying TNFα [6, 8, 34]. Although inducing EndMT in endocardial spheres was difficult, we successfully verified that 3D-derived VELCs can undergo EndMT using an alternative 2D method, achieving a conversion rate of about 20% from CDH5^+^/ACTA2^−^ VELCs to CDH5^−^/ACTA2^+^ VILCs, which is comparable to other published results after EndMT induction [6, 8]. Generally, the transdifferentiation rate after EndMT induction was higher in VELCs derived from 2D differentiation (≈ 48 ACTA2^+^ cells) than in 3D-derived VELCs (≈ 20%), which could be related o the greater maturity of VELCs derived from 3D endocardial spheres, as well as to the different growth factor combinations used in 2D (FGF2 + TGFβ1) and 3D (only TGFβ1). We assumed that EndMT induced by TGFβ1 alone would be more efficient than that induced by a combination of FGF2 and TGFβ1 because FGF2 was reported to affect TGFBR1 expression [35] and EndMT induction [36], but our results did not reflect this. Some publications have shown that hiPSC-derived endothelial cells (ECs) exhibit functional deficits relative to established EC lines such as HUVECs and/or show lower expression of *VWF* and *NOS3*, indicating a premature phenotype [35, 36]. Our results suggest that 3D differentiation methods may produce more mature VELCs, as we observed higher expression of *VWF* and *NOS3* compared to cells differentiated in a 2D monolayer. However, he 2D and 3D protocols not only differ in the “dimensions” used for differentiation but also in the growth factors and small molecules applied, which might affect cell maturity and their ability to undergo EndMT. Since 2D-and 3D-derived VELCs both arise from CPCs as an intermediate state and have been analyzed at the same time points, we suspect that the observed differences in maturity are mainly due to three-dimensional differentiation, which provides a more physiological environment than monolayer differentiation.

## Conclusion

In summary, we have developed a 3D in vitro differentiation protocol using spheres to efficiently generate VELCs. The differentiation protocol is relatively straightforward and suitable for upscaling. Characterization of these 3D differentiated VELCs indicates, based on *VWF* and *NOS3* expression patterns, that they are more mature than the 2D differentiated cells. An additional benefit of our protocol is the use of small molecules, which lowers the costs compared to growth factor-based protocols, and the adoption of RPMI and E8 media, which are well-characterized and available in standardized formulations.

## Supplementary Information

Below is the link to the electronic supplementary material.

Supplementary Material 1.

## Data Availability

The raw data from bulk RNA-Seq analysis are available on the Sequence Read Archive (SRA) data platform, accession number PRJNA1411047. The datasets generated and/or analysed during the current study are not yet publicly available due, but are available from the corresponding author on reasonable request.

## Abbreviations

2D: Two-dimensional
3D: Three-dimensional
AVS: Aortic valve stenosis
BMP4: Bone morphogenetic protein 4
BSA: Bovine serum albumin
CP: Cardiac progenitor
CPC: Cardiac progenitor cell
DEG: Differentially expressed gene
DPBS: Dulbecco’s balanced salt solution
E6: Essential 6^TM^ (medium)
E8: Essential 8^TM^ (medium)
EDTA: Ethylendiaminetetraacetic acid
EndMT: Endothelial-to-mesenchymal transition
FGF2: Fibroblast growth factor 2
GiWi: GSK inhibition/Wnt inhibition
GO: Gene ontology
GSEA: Gene set enrichment analysis
GSK: Glycogen sythase
hiPSCs: Human induced pluripotent stem cell
LDL: Low density lipoprotein
log2(FC): log2(fold change)
MACS: Magnet-assisted cell sorting
RPK: Reads per kilobase
RPMI: Roswell Park Memorial Institute (medium)
TGFβ1: Transfroming growth factor β 1
TPM: Transcripts per million
VEC: (Adult) valve endothelial cell
VEGF: Vascular endothelial growth factor
VELC: (hiPSC-derived) valve endothelial-like cell
VIC: (Adult) valve interstitial cell
VILC: (hiPSC-derived) Valve interstitial-like cell

## References

[1] Martinsson A, Li X, Andersson C, Nilsson J, Smith JG, Sundquist K. Temporal trends in the incidence and prognosis of aortic stenosis: a nationwide study of the Swedish population. Circulation. 2015;131(11):988–94. 25779541 10.1161/CIRCULATIONAHA.114.012906

[2] Chen Y, Yiu K-H. Growing importance of valvular heart disease in the elderly. J Thorac Dis. 2016;8(12):E1701–3. 28149618 10.21037/jtd.2016.12.23 PMC5227228

[3] Butcher JT, Nerem RM. Valvular endothelial cells and the mechanoregulation of valvular pathology. Philos Trans R Soc Lond B Biol Sci. 2007;362(1484):1445–57. 17569641 10.1098/rstb.2007.2127 PMC2440407

[4] Bao X, Bhute VJ, Han T, Qian T, Lian X, Palecek SP. Human pluripotent stem cell-derived epicardial progenitors can differentiate to endocardial-like endothelial cells. Bioeng Transl Med. 2017;2(2):191–201. 29170757 10.1002/btm2.10062 PMC5675097

[5] Neri T, Hiriart E, van Vliet PP, Faure E, Norris RA, Farhat B, et al. Human pre-valvular endocardial cells derived from pluripotent stem cells recapitulate cardiac pathophysiological valvulogenesis. Nat Commun. 2019;10(1):1929. 31028265 10.1038/s41467-019-09459-5 PMC6486645

[6] Mikryukov AA, Mazine A, Wei B, Yang D, Miao Y, Gu M, et al. BMP10 Signaling Promotes the Development of Endocardial Cells from Human Pluripotent Stem Cell-Derived Cardiovascular Progenitors. Cell Stem Cell. 2021;28(1):96–e1117. 33142114 10.1016/j.stem.2020.10.003

[7] Cheng L, Xie M, Qiao W, Song Y, Zhang Y, Geng Y, et al. Generation and characterization of cardiac valve endothelial-like cells from human pluripotent stem cells. Commun Biol. 2021;4(1):1039. 34489520 10.1038/s42003-021-02571-7 PMC8421482

[8] Liu CZ, Prasad A, Jadhav B, Liu Y, Gu M, Sharp AJ, et al. Feeder-free generation and characterization of endocardial and cardiac valve cells from human pluripotent stem cells. iScience. 2024;27(1):108599. 38170020 10.1016/j.isci.2023.108599 PMC10758960

[9] Cai Z, Zhu M, Xu L, Wang Y, Xu Y, Yim WY, et al. Directed Differentiation of Human Induced Pluripotent Stem Cells to Heart Valve Cells. Circulation. 2024;149(18):1435–56. 38357822 10.1161/CIRCULATIONAHA.123.065143 PMC11062615

[10] Cai S, Fu X, Sheng Z, Dedifferentiation. A New Approach in Stem Cell Research. Bioscience. 2007;57(8):655–62.

[11] Urzì O, Gasparro R, Costanzo E, de Luca A, Giavaresi G, Fontana S, et al. Three-dimensional cell cultures: the bridge between in vitro and in vivo models. Int J Mol Sci. 2023;24(15):12046. 37569426 10.3390/ijms241512046 PMC10419178

[12] Fonoudi H, Ansari H, Abbasalizadeh S, Larijani MR, Kiani S, Hashemizadeh S, et al. A Universal and Robust Integrated Platform for the Scalable Production of Human Cardiomyocytes From Pluripotent Stem Cells. Stem Cells Transl Med. 2015;4(12):1482–94. 26511653 10.5966/sctm.2014-0275 PMC4675501

[13] Afgan E, Baker D, Batut B, van den Beek M, Bouvier D, Cech M, et al. The Galaxy platform for accessible, reproducible and collaborative biomedical analyses: 2018 update. Nucleic Acids Res. 2018;46(W1):W537–44. 29790989 10.1093/nar/gky379 PMC6030816

[14] Dobin A, Davis CA, Schlesinger F, Drenkow J, Zaleski C, Jha S, et al. STAR: ultrafast universal RNA-seq aligner. Bioinformatics. 2013;29(1):15–21. 23104886 10.1093/bioinformatics/bts635 PMC3530905

[15] Liao Y, Smyth GK, Shi W. featureCounts: an efficient general purpose program for assigning sequence reads to genomic features. Bioinformatics. 2014;30(7):923–30. 24227677 10.1093/bioinformatics/btt656

[16] Love MI, Huber W, Anders S. Moderated estimation of fold change and dispersion for RNA-seq data with DESeq2. Genome Biol. 2014;15(12):550. 25516281 10.1186/s13059-014-0550-8 PMC4302049

[17] Misfeldt AM, Boyle SC, Tompkins KL, Bautch VL, Labosky PA, Baldwin HS. Endocardial cells are a distinct endothelial lineage derived from Flk1 + multipotent cardiovascular progenitors. Dev Biol. 2009;333(1):78–89. 19576203 10.1016/j.ydbio.2009.06.033

[18] Ma J, Sanchez-Duffhues G, Goumans M-J, Dijke P. ten. TGF-β-Induced Endothelial to Mesenchymal Transition in Disease and Tissue Engineering. Front Cell Dev Biol. 2020;8:260. 32373613 10.3389/fcell.2020.00260 PMC7187792

[19] Lu X, Gong J, Dennery PA, Yao H. Endothelial-to-mesenchymal transition: Pathogenesis and therapeutic targets for chronic pulmonary and vascular diseases. Biochem Pharmacol. 2019;168:100–7. 31251941 10.1016/j.bcp.2019.06.021 PMC6733623

[20] Lian X, Zhang J, Azarin SM, Zhu K, Hazeltine LB, Bao X, et al. Directed cardiomyocyte differentiation from human pluripotent stem cells by modulating Wnt/β-catenin signaling under fully defined conditions. Nat Protoc. 2013;8(1):162–75. 23257984 10.1038/nprot.2012.150 PMC3612968

[21] Chong JJH, Reinecke H, Iwata M, Torok-Storb B, Stempien-Otero A, Murry CE. Progenitor cells identified by PDGFR-alpha expression in the developing and diseased human heart. Stem Cells Dev. 2013;22(13):1932–43. 23391309 10.1089/scd.2012.0542 PMC3685392

[22] Yilbas AE, Hamilton A, Wang Y, Mach H, Lacroix N, Davis DR, et al. Activation of GATA4 gene expression at the early stage of cardiac specification. Front Chem. 2014;2:12. 24790981 10.3389/fchem.2014.00012 PMC3982529

[23] Chen G, Gulbranson DR, Hou Z, Bolin JM, Ruotti V, Probasco MD, et al. Chemically defined conditions for human iPS cell derivation and culture. Nat Methods. 2011;8(5):424–9. 21478862 10.1038/nmeth.1593 PMC3084903

[24] Prummel KD, Nieuwenhuize S, Mosimann C. The lateral plate mesoderm. Development. 2020;147(12):dev175059. 32561665 10.1242/dev.175059 PMC7328003

[25] Morita Y, Tohyama S. Metabolic Regulation of Cardiac Differentiation and Maturation in Pluripotent Stem Cells: A Lesson from Heart Development. JMA J. 2020;3(3):193–200. 33150253 10.31662/jmaj.2020-0036 PMC7590396

[26] Dor Y, Camenisch TD, Itin A, Fishman GI, McDonald JA, Carmeliet P, et al. A novel role for VEGF in endocardial cushion formation and its potential contribution to congenital heart defects. Development. 2001;128(9):1531–8. 11290292 10.1242/dev.128.9.1531

[27] Ergir E, La Oliver-De Cruz J, Fernandes S, Cassani M, Niro F, Pereira-Sousa D, et al. Generation and maturation of human iPSC-derived 3D organotypic cardiac microtissues in long-term culture. Sci Rep. 2022;12(1):17409. 36257968 10.1038/s41598-022-22225-w PMC9579206

[28] Suominen S, Hyypijev T, Venäläinen M, Yrjänäinen A, Vuorenpää H, Lehti-Polojärvi M, et al. Improvements in maturity and stability of 3D iPSC-derived hepatocyte-like cell cultures. Cells. 2023;12(19):2368. 37830581 10.3390/cells12192368 PMC10571736

[29] de Leeuw SM, Davaz S, Wanner D, Milleret V, Ehrbar M, Gietl A, et al. Increased maturation of iPSC-derived neurons in a hydrogel-based 3D culture. J Neurosci Methods. 2021;360:109254. 34126141 10.1016/j.jneumeth.2021.109254

[30] Sam J, Pampiermole R, Broersen K, Das R. Upscaling and differentiating human induced pluripotent stem cell spheroids in 3D. Cytotherapy. 2024;26(6):S222–3.

[31] Wu B, Baldwin HS, Zhou B. Nfatc1 directs the endocardial progenitor cells to make heart valve primordium. Trends Cardiovasc Med. 2013;23(8):294–300. 23669445 10.1016/j.tcm.2013.04.003 PMC3766465

[32] O’Donnell A, Yutzey KE. Mechanisms of heart valve development and disease. Development. 2020;147(13):dev183020. 32620577 10.1242/dev.183020 PMC7338271

[33] Bischoff J, Casanovas G, Wylie-Sears J, Kim DH, Bartko PE, Guerrero JL, et al. CD45 Expression in Mitral Valve Endothelial Cells After Myocardial Infarction. Circul Res. 2016;119(11):1215–25. 10.1161/CIRCRESAHA.116.309598 PMC5215059 27750208

[34] Nehl D, Goody PR, Maus K, Pfeifer A, Aikawa E, Bakthiary F, et al. Human and porcine aortic valve endothelial and interstitial cell isolation and characterization. Front Cardiovasc Med. 2023;10:1151028. 37408661 10.3389/fcvm.2023.1151028 PMC10318150

[35] Bezenah JR, Kong YP, Putnam AJ. Evaluating the potential of endothelial cells derived from human induced pluripotent stem cells to form microvascular networks in 3D cultures. Sci Rep. 2018;8(1):2671. 29422650 10.1038/s41598-018-20966-1 PMC5805762

[36] Rieck S, Sharma K, Altringer C, Hesse M, Triantafyllou C, Zhang Y, et al. Forward programming of human induced pluripotent stem cells via the ETS variant transcription factor 2: rapid, reproducible, and cost-effective generation of highly enriched, functional endothelial cells. Cardiovasc Res. 2024;120(12):1472–84. 38916487 10.1093/cvr/cvae129

