# Supplementary material for: Differentiation of human induced pluripotent stem cells into cardiac valve cells using 2D and 3D differentiation protocols
Source: Stem Cell Res Ther. 2026 Jul 2;17:239. doi: 10.1186/s13287-026-05132-z (PMC13330356; doi:10.1186/s13287-026-05132-z)
Supplement: Supplementary file 1 — Supplementary Material 1. [file 13287_2026_5132_MOESM1_ESM.pdf]

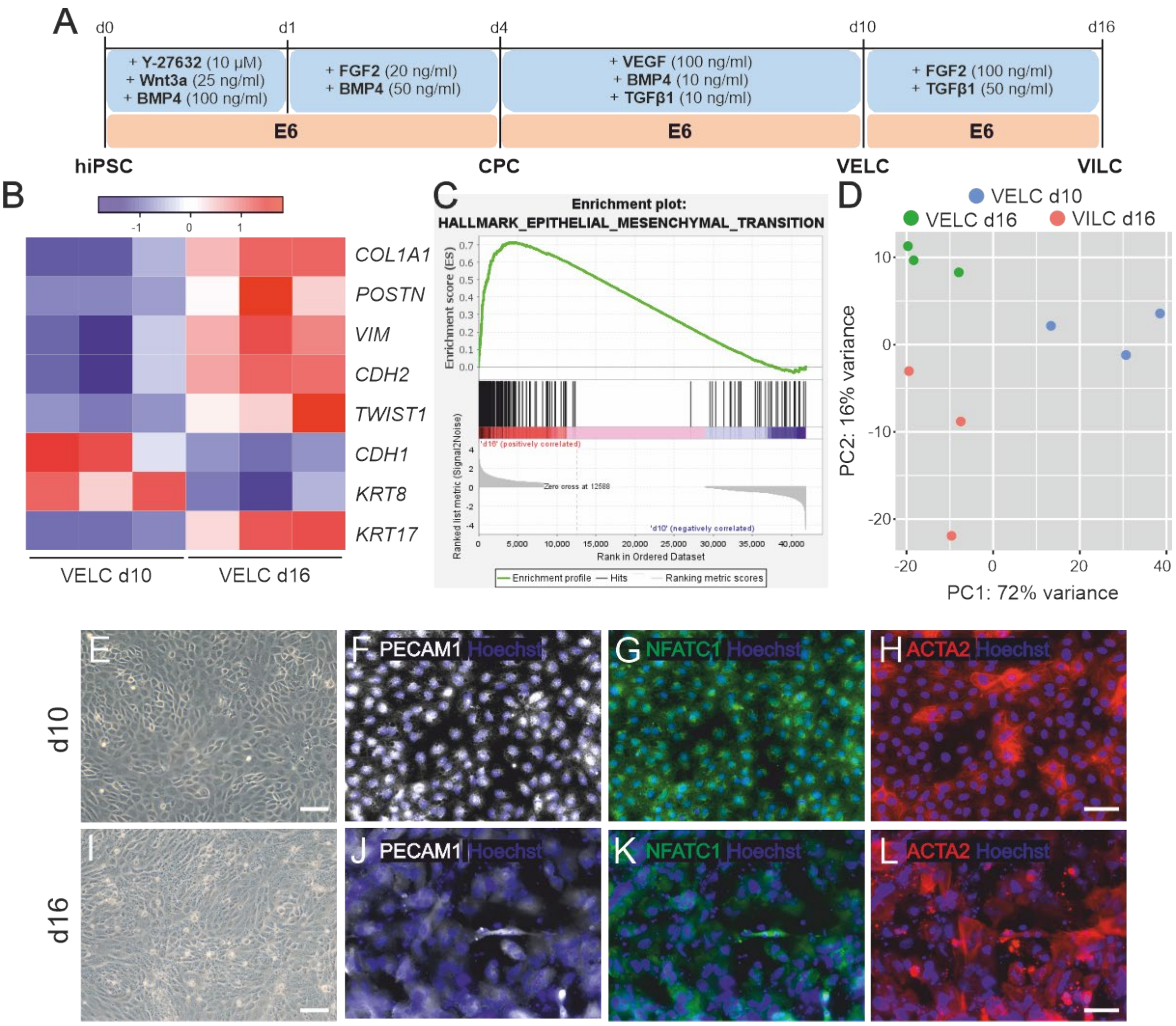

Suppl. Figure 1: Monolayer differentiation (2D) protocol for endocardial cells originally published by Cheng et al. in Communications Biology in 2021. (A) Human-induced pluripotent stem cells (hiPSCs) were first differentiated into cardiac progenitor cells (CPCs), then further specified into valve endothelial-like cells (VELCs). (B) Heatmap of mesenchymal and epithelial marker expression in VELCs at day 10 and day 16. (C) Gene set enrichment analysis (GSEA, hallmark gene set) showed upregulation of genes correlated with epithelial-to-mesenchymal transition in VELCs at day 16. (D) Principal component analysis (PCA) based on bulk RNA-Seq data for VELC at day 10 and 16 and VILCs at day 16. (E-H) Phase contrast image (E) and immunofluorescence staining (F-H) of VELCs at day 10 differentiated with the original 2D protocol; scale bar = 50 µm. (I-L) Phase contrast image (I) and Immunofluorescence staining (J-L) of VELCs at day 16 differentiated with the original 2D protocol; scale bar = 50 µm. For heatmaps only differentially expressed genes with a log2(FC) > 1 and an adj. p-value < 0.05 have been considered.

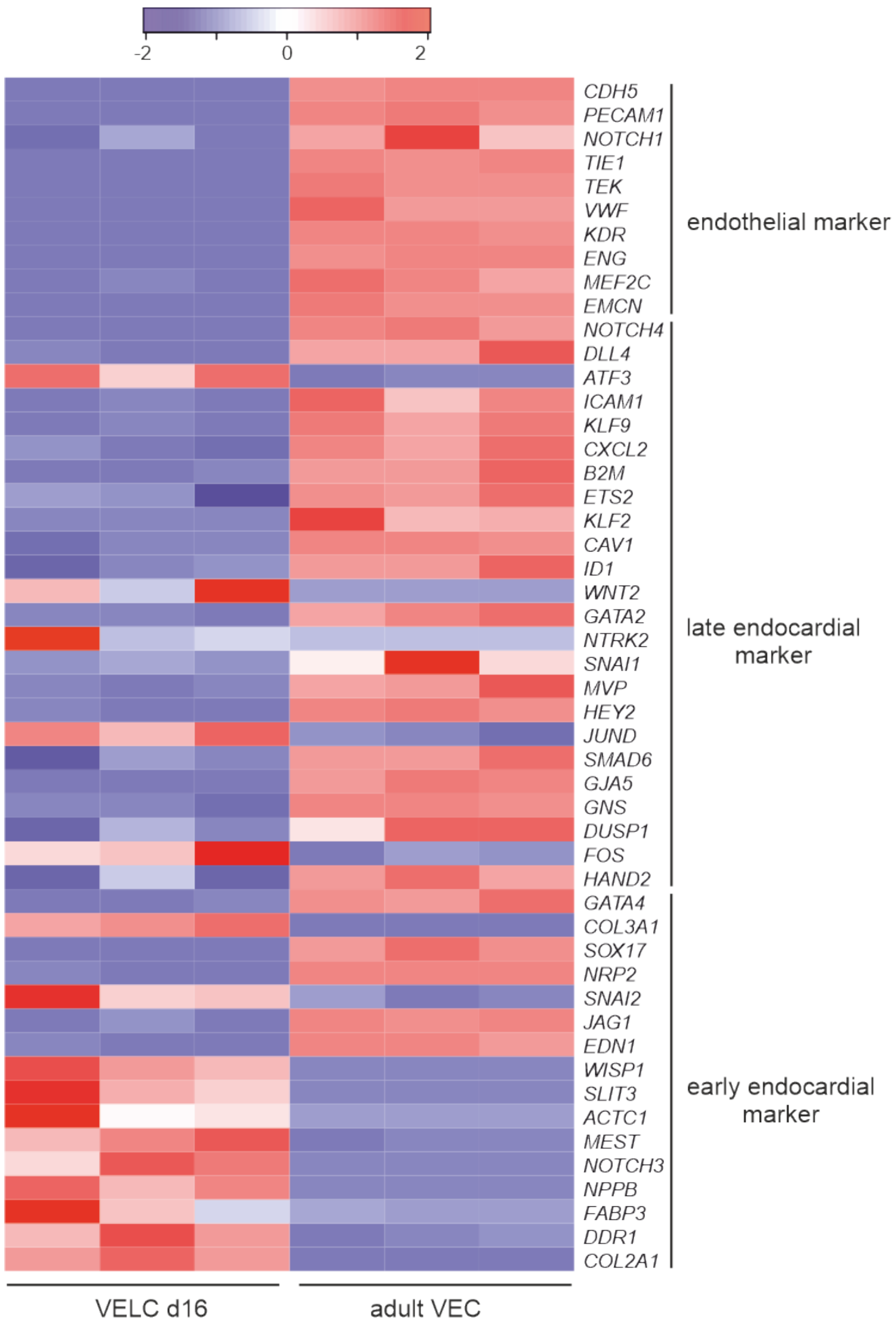

Suppl. Figure 2: Comparison of a gene panel including a general endothelial marker and markers for early- and late-stage endocardial cells. 2D VELCs differentiated using the improved protocol have been compared with adult VECs for expression of endothelial markers and early- and late-stage endocardial marker genes. The gene panel was originally established by Cheng et al. For heatmaps, only differentially expressed genes with a log2(FC) > 1 and an adj. p-value < 0.05 have been considered.

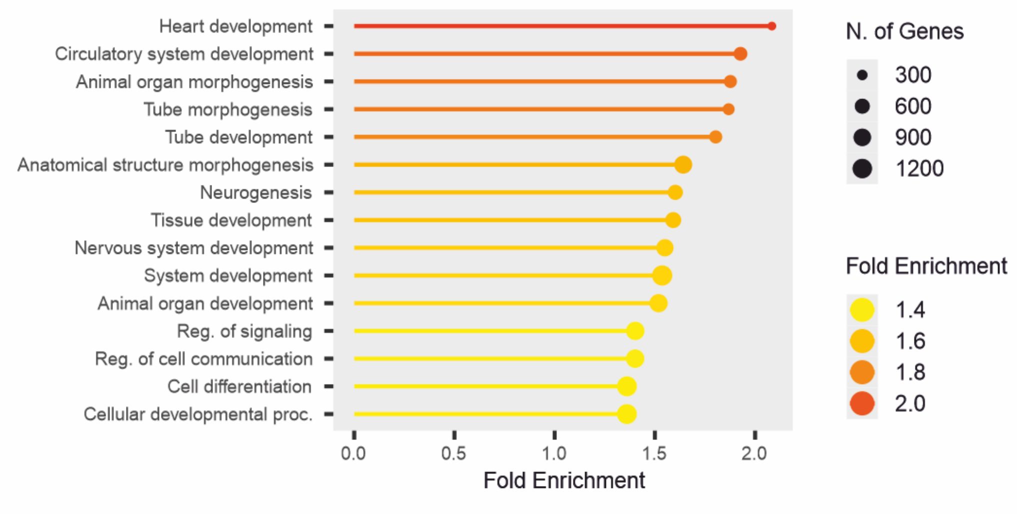

Suppl. Figure 3: Comparison of 3D CP spheres on day 4 with undifferentiated hiPSC spheres. Gene Ontology (GO) analysis of upregulated genes in CP spheres on day 4 compared with hiPSC spheres on day 0 of 3D differentiation, presented as a fold enrichment plot.

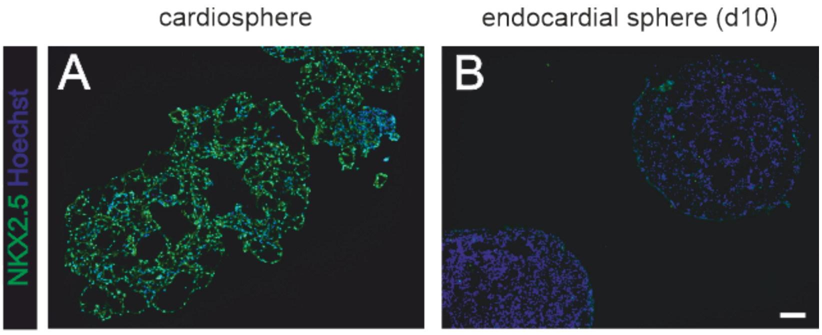

Suppl. Figure 4: Nkx2.5 expression in different 3D spheres. (A) beating cardiospheres (positive control), (B) endocardial spheres at day; scale bar = 100 µm.

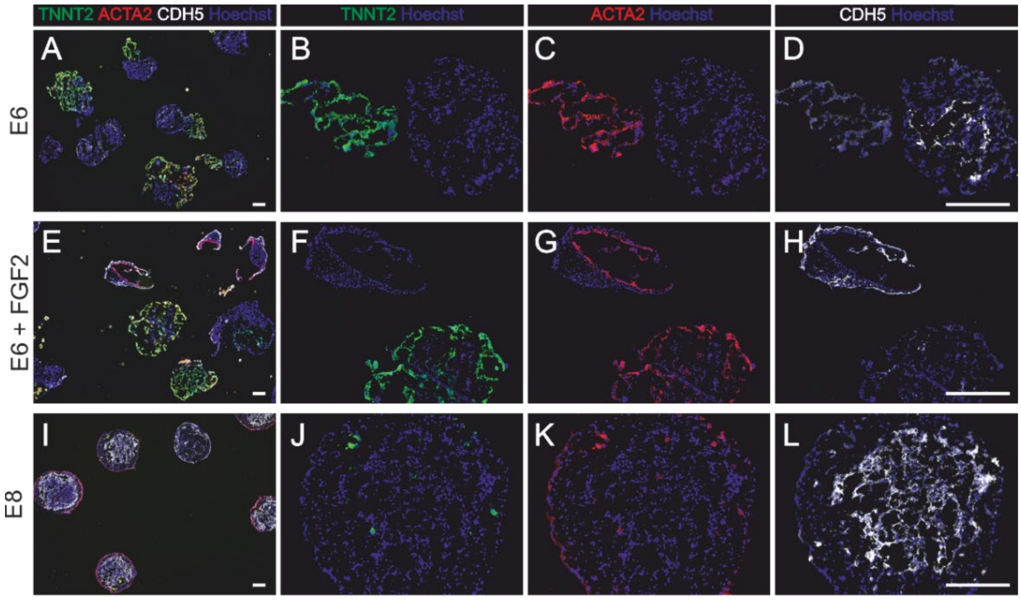

Suppl. Figure 5: Effect of FGF2 on endocardial differentiation. (A-L) Immunofluorescence staining of endocardial spheres differentiated for 6 days in E6 medium (A-D), E6 medium supplemented with 50 ng/ml FGF2 (E-H) or E8 medium (I-L); scale bar = 200 µm.

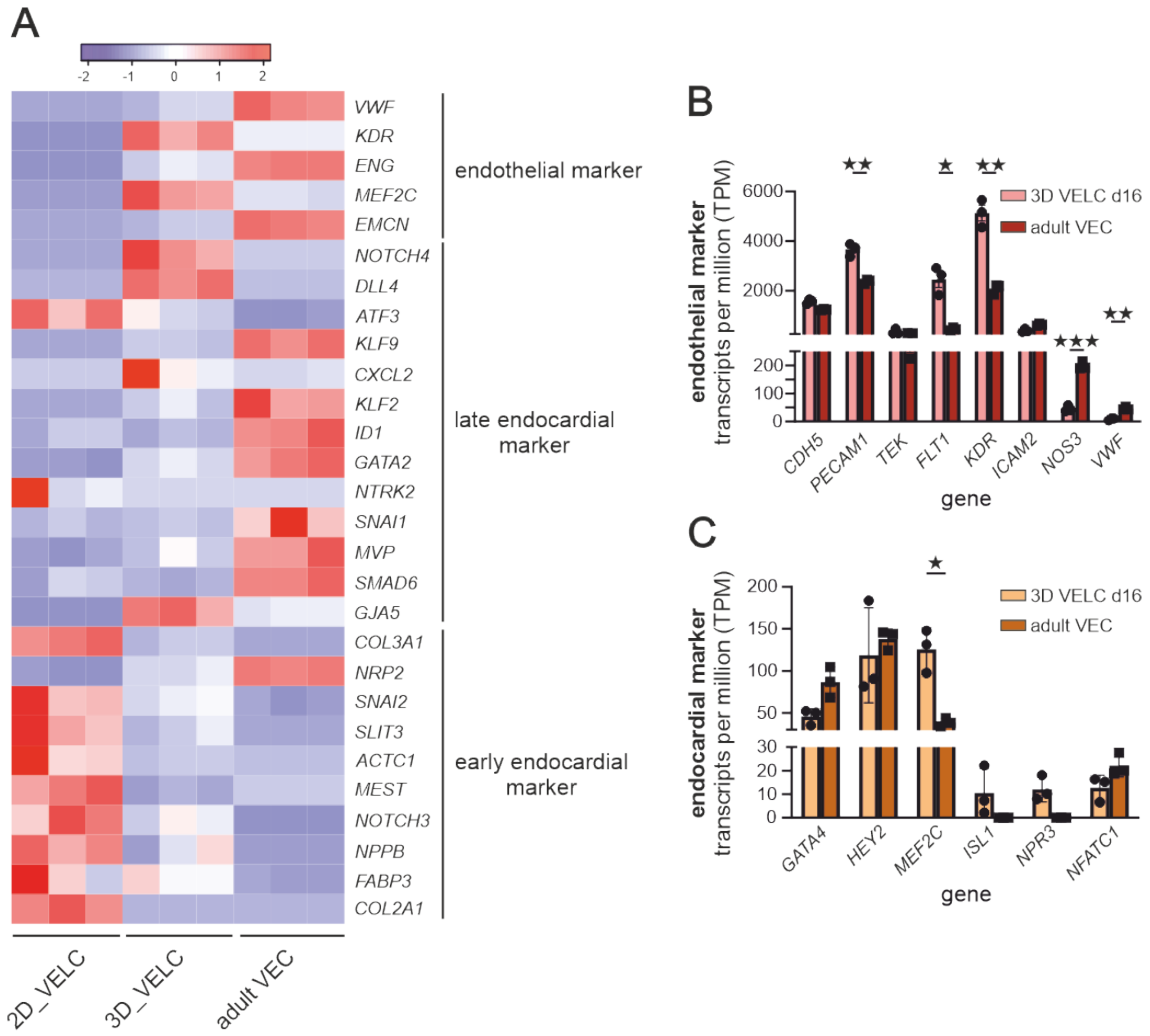

Suppl. Figure 6: Comparison of a gene panel including general endothelial markers as well as markers for early and late-stage endocardial cells in 2D VELCs, 3D VELCs, and adult VECs. 2D VELCs differentiated using the improved protocol have been compared with 3D-derived VELCs and adult VECs for expression of endothelial markers and early- and late-stage endodermic marker genes. The gene panel used was originally reported by Cheng et al. **(B, C)** Transcripts per million (TPM) were calculated for general endothelial markers **(B)** or endocardial markers **(C)** to compare 3D VELCs with adult VECs. For heatmaps, only differentially expressed genes with a log2(FC) > 1 and an adj. p-value < 0.05 have been considered.

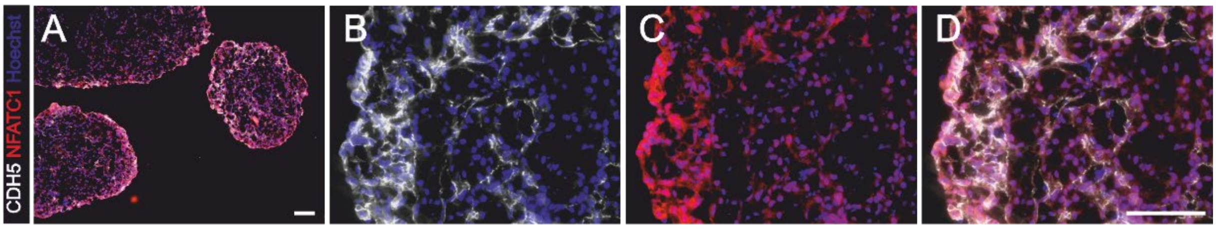

Suppl. Figure 7: Validation of 3D differentiation protocol in the C133bm-s4 hiPSC line. (A-C) Immunofluorescence staining for Cd144 and NFATc1 in endocardial spheres at day 10 (d10) of differentiation. (A) Overview image; scale bar = 100 µm. (B-D) Close-up images; scale bar = 100 µm.

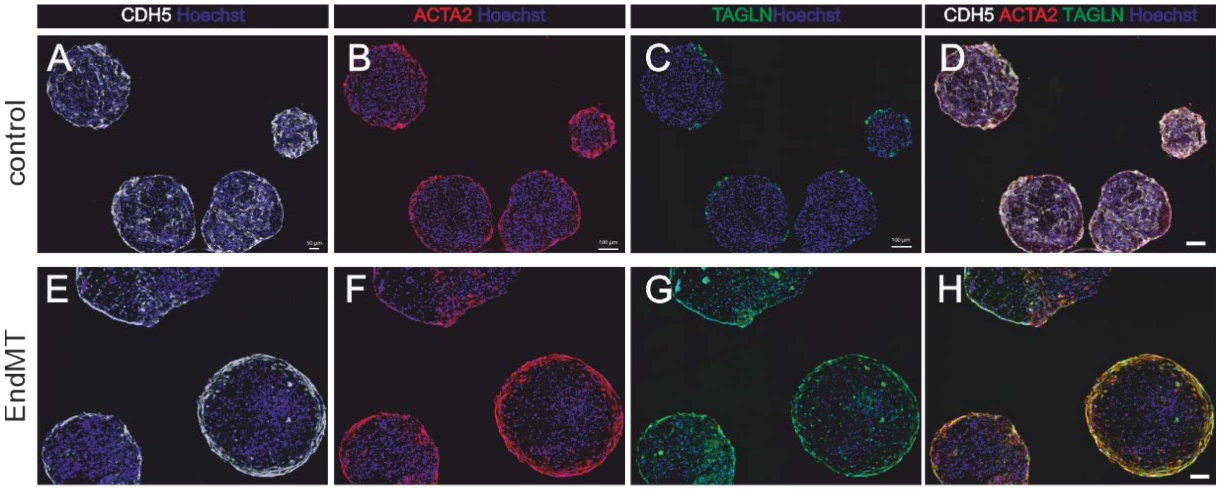

Suppl. Figure 8: EndMT induction in endocardial spheres. (A-D) Immunofluorescence staining of endocardial spheres at day 16 (d16); scale bar = 100 µm. (E-H) Immunofluorescence staining of endocardial spheres at d16 after six days of EndMT induction with 200 ng / ml TGFβ1; scale bar = 100 µm.
